# Estimating the motor exploration in reinforcement learning

**DOI:** 10.1016/j.isci.2025.114398

**Published:** 2025-12-09

**Authors:** Anja T. Zai, Corinna Lorenz, Shakana Srikantharajah, Nicolas Giret, Richard H.R. Hahnloser

**Affiliations:** 1 Institute of Neuroinformatics, University of Zurich and ETH Zurich, Zurich, Switzerland; 2 Neuroscience Center Zurich (ZNZ), University of Zurich and ETH Zurich, Zurich, Switzerland; 3 Institut des Neurosciences Paris Saclay, UM CNRS, Université Paris Saclay, Saclay in Île-de-France, France

**Keywords:** neuroscience, sensory neuroscience, cognitive neuroscience

## Abstract

What exploration strategies do animals use to learn motor skills? Reinforcement learning (RL) theory is a powerful framework to study motor learning, but provides no guidance for estimating motor exploration – the behavioral component aimed at discovering better strategies. We address this gap by taking inspiration from the brain’s modular organization and postulating a latent learner that explores by injecting an additive source of ideal randomness into behavior. Assuming the learner is ignorant of other motor components, evolutionary fitness argues that these should display mainly non-ideal variability.

We verify this behavioral decomposition in songbirds undergoing vocal pitch conditioning. The estimated vocal explorations account for the motor contribution of a cortico-basal ganglia pathway, while other components capture birds’ suboptimal learning trajectories. Latent RL therefore provides a normative improvement over classical RL, making exploration explicit and suggesting that evolutionary pressure favors the randomness of exploration over strict behavioral optimality.

## Highlights

• We introduce a latent RL agent that learns optimally from its exploration
• Latent RL agrees with non-optimal learning trajectories in songbirds and humans
• Explorations can be estimated from behavior on a single-trial basis
• Estimated explorations match brain-generated motor variability driving learning

## Graphical abstract

**Figure:**
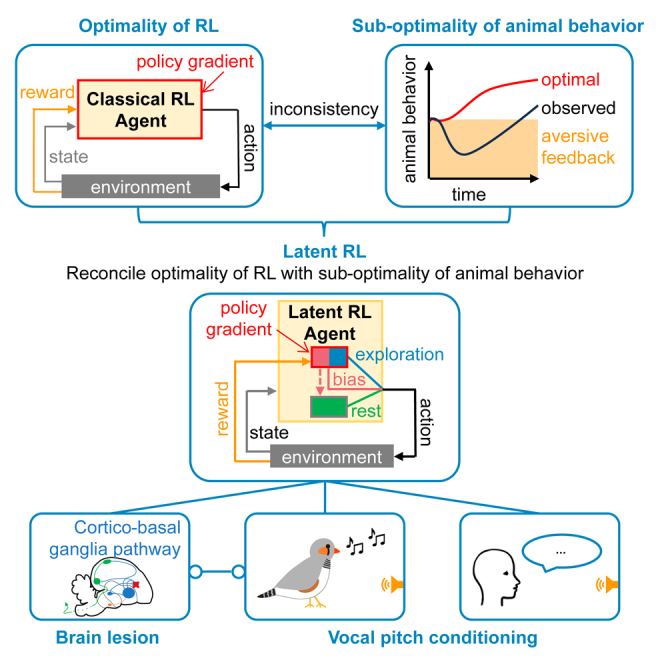

## Introduction

Reinforcement learning (RL) is a computational theory about identifying optimal behavior that maximizes the collected reward.^1^^,^^2^^,^^3^ RL is a successful strategy for gaming^4^^,^^5^ and holds promise as a theoretical framework for understanding neural processing, particularly in dopamine neurons,^6^^,^^7^^,^^8^ but see.^9^ Even though early concepts of RL were inspired by animal behavior,^10^ applying RL to natural behaviors has remained challenging. Mainly, behavior tends to be sub-optimal, violating optimal action policies.^11^^,^^12^^,^^13^ Behavioral sub-optimality by itself does not falsify RL theory; it is the overestimated amount of exploration that remains perplexing.

Exploration is a central component of RL; it is conceived as actions that deliberately deviate from an optimal strategy in the hope of finding a better strategy leading to more reward, also known as the exploration-exploitation tradeoff. RL theory applies to the entire range from pure exploitation to pure exploration,^14^ and so there are virtually limitless ways of introducing exploration into RL models, i.e., the theory is under-constrained and offers no principled way of estimating the behavioral component attributable to exploration. Despite this modeling freedom, when fitting behavior with normative RL models that incorporate an exploitation-exploration tradeoff, subjects seem to learn less from deviant behavioral variants than they theoretically could,^15^^,^^16^ which means that some of their behavioral variability stems not from decisions to explore.^17^

Indeed, there is growing evidence that the brain is capable of learning from only a part of motor variability^18^^,^^19^ and that some sources of motor variability are disconnected from a learning experience.^19^ For example, motor noise arising in the peripheral motor system is thought to provide little benefit for improving behavioral output,^20^ and artificially imposed motor noise is of no benefit to subjects for increasing the success of their actions.^21^^,^^22^ A behavior-congruent RL theory, therefore, needs to account for more than just the exploitation of good strategies and exploration of new strategies.

**Figure 1:**
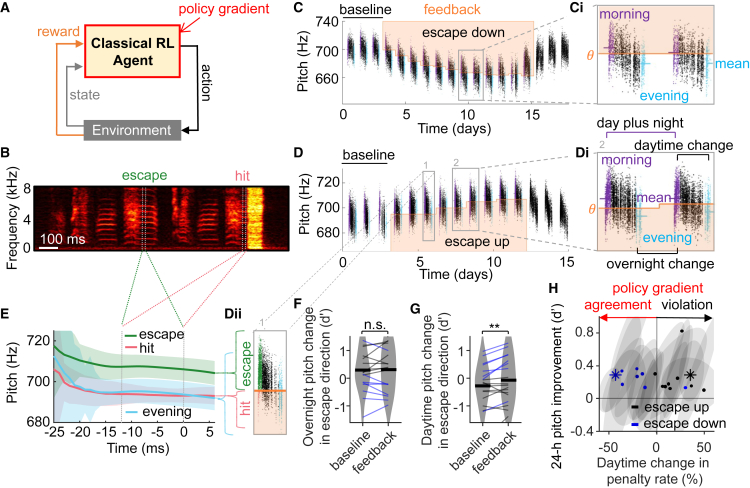
Classical RL clashes with non-monotonic learning trajectories (A) In classical RL, an agent potentially modifies all behaviors to steadily increase reward — the agent senses the entire policy gradient. (B) Song spectrogram of a bird subjected to aversive WN stimuli (hit, yellow area) contingent on low-pitch syllables. The pitch measurement window is indicated by vertical white dotted lines. (C and D) Pitches (dots) of the targeted syllable before (baseline), during, and after contingent WN feedback (the orange line and shaded area delimit the pitch threshold *θ* and pitch range of WN feedback). (Ci and Di) Evening pitches (blue dots) are lower than morning pitches (purple dots) despite the aversive WN stimuli they trigger in Di (first/last hour, respectively). Dii Evening pitches (blue) are closer to WN-eliciting pitches in the morning (hits, red), than to WN-escaping pitches (green). (E) Average pitch trace as a function of time in the syllable (±1 standard deviation (SD) in shading). The average evening pitch trace is similar to the average trace of previously penalized (hit) syllables. (F) Average overnight pitch changes (thick black line) in the escape direction (d’>0) were similar during the baseline period and the feedback period. Nearly all birds (thin lines) maintained the sign of their overnight pitch change, regardless of whether their penalized pitch zone was low (black) or high (blue). The only bird that changed the sign of overnight pitch changes did so in the penalized direction (d’<0) rather than the escape direction. (G) During the feedback period, average daytime pitch changes increased in the escape direction relative to baseline (∗∗*p* < 0.01, paired *t* test, n = 18 birds). (H) In half of the birds (arrow labeled violation), the penalty rate (x axis) increased during the day although all birds improved their pitch (away from the penalized zone) over the course of 24-h periods (y axis). Dots represent single birds (blue, escaped down; black, escaped up), and shaded ellipses represent 1 SD. Birds shown in C and D are marked with blue and black asterisks.

To keep the mathematical rigor of RL (Figure 1A) but to make it amenable to natural behaviors and their hidden explorative components, we abandon the overall behavioral optimality as a goal of RL. Instead, we frame RL as a model of a hypothesized latent learner, a behavioral module that learns from its random perturbations of behavioral output. Such a behavioral module dedicated to explorative motor variability has been hypothesized^23^^,^^24^ and it encapsulates that many skills are learned and recalled in separate brain regions.^25^^,^^26^^,^^27^^,^^28^^,^^29^ To formulate latent RL as a normative principle suitable for statistical inference,^30^ we assume that the latent learner maximally learns from the effects of its explorations on reward, whereas all other modules are unable to meaningfully integrate reward experience on a trial-by-trial basis.

We assume that the latent explorations exclusively form an ideal source of randomness, i.e., the latent learner alone (exclusively) contributes to behavioral trials in terms of independent and identically distributed (iid, ideal) random explorations. We motivate this assumption of ideal exploration with computational generality and with evolutionary fitness: On the one hand, random sampling is the basis of science as it guarantees the collection of unbiased data (randomness in clinical trials avoids spurious correlations with uncontrolled influences; and, in games such as rock, scissors, and paper, random strategies make a player robust to adversarial counter strategies). On the other hand, the “exclusivity” attribute, i.e., that no other module contributes iid additive motor variability, maximizes the agent’s learning efficiency (the effect size in clinical trials decreases under ignorance whether some subjects were administered a treatment or a placebo).

We apply this idea to behavioral data from songbirds that we instrumentally condition to change their song away from the stable version they acquired from a tutor,^31^ a faculty that depends on a cortico-basal ganglia (CBG) loop^25^^,^^26^^,^^32^^,^^33^^,^^34^^,^^35^ and its output, the lateral magnocellular nucleus of the anterior nidopallium (LMAN).

First, we show the failure of classical RL models: many birds do not behave optimally in that their circadian singing patterns paradoxically appear to counteract the daytime-learned motor changes. Because we find that birds do not learn overnight either, we propose that songbirds reinforce merely ideal explorative motor perturbations rather than all behavioral output. This model produces excellent fits of behavioral learning trajectories, and, the ablation of the exploration module in our model matches the effects of LMAN lesions. We probe for the generality of the proposed reinforcement mechanism by subjecting humans to a similar conditioning task.

## Results

After letting birds freely sing for a few days (baseline period), we applied a widely used instrumental conditioning paradigm (feedback period) to train zebra finches (*Taeniopygia guttata*) to modify the pitch (fundamental frequency) of a chosen target syllable.^31^ As a conditioning stimulus, we played a loud white-noise (WN) sound through a loudspeaker when the pitch of the target syllable was below (or above) a set pitch threshold, Figure 1B (see STAR Methods “song recording and pitch conditioning in birds”). We set the threshold every night based on the pitch of the day before. Across days, birds cumulatively adapted the pitch of the targeted syllable either down (Figure 1C) or up (Figure 1D) to try to escape the aversive stimuli and against their tendency to maintain a stable pitch.

### Behavioral incongruences of classical reinforcement learning

During the baseline period, we noticed that birds’ pitch drifted with circadian rhythmicity, similar to earlier reports.^36^^,^^37^^,^^38^ The 18 birds on average decreased their pitch during the day from morning to evening (*d*′ = -0.48 ± 0.69, range −2.3 to 1.2, *p* = 0.009, df = 17, tstat = −2.9, two-tailed *t* test). Their circadian pitch pattern was accompanied by counteracting overnight pitch discontinuities (*d*′ = 0.49 ± 0.71, range −1.4 to 1.9, *p* = 0.01, df = 17, tstat = 2.9, two-tailed *t* test) with the overall effect of them maintaining a stable average pitch across baseline days.

During the feedback period, overnight pitch discontinuities did not change (*d*′ = 0.009 ± 0.34 in the escape direction, *p* = 0.91, df = 17, tstat = 0.12, two-tailed *t* test, Figure 1F). In contrast, daytime pitch changes increased or decreased to counteract the aversive stimuli (*d*′ = 0.22 ± 0.31 in the escape direction, *p* = 0.009, df = 17, tstat = 2.9, two-tailed *t* test, Figure 1G). All birds shifted pitch to escape the aversive stimuli (Figure 1H), but the strong circadian pattern caused many birds to receive more aversive stimuli in the evening than in the morning (example in Figure 1Di).

Such non-monotonicity of motor learning clashes with earlier conclusions that learning results from the reinforcement of successful past actions,^33^ since many birds in our study seemingly just did the opposite (Figures 1Dii and 1E): to selectively repeat the penalized syllable variants (rather than the unpenalized ones).

In total, *n* = 9/18 birds coped with the tradeoff to maintain a stable baseline song and to escape the penalty by violating traditional notions of RL: they changed their pitch during the day to collect more penalties, not fewer (*p* < 0.05, one-tailed *t* test of a higher fraction of syllables penalized in the evening than in the morning). Their pitch adaptation away from the penalized zone was apparent only over the course of 24-h periods (Figure 1H). In other words, these birds did not exhibit a circadian pattern that aligned with the escape direction (Figure 1C), but their circadian pattern acted against the escape direction (Figure 1D). As a result, these birds appeared not to follow the presumed “policy gradient” of decreasing the punishment rate during the day (Figure 1H)^1^^,^^2^^,^^3^; and instead, they violated the policy gradient assumption by increasing the punishment rate (Figures 1E and 1H). Because our data show no evidence for overnight learning either (to the contrary, Figure 1F), these observations challenge classical RL models of reward maximization (or punishment minimization) and call for a revision of birdsong RL models.

### Latent reinforcement learning

**Figure 2:**
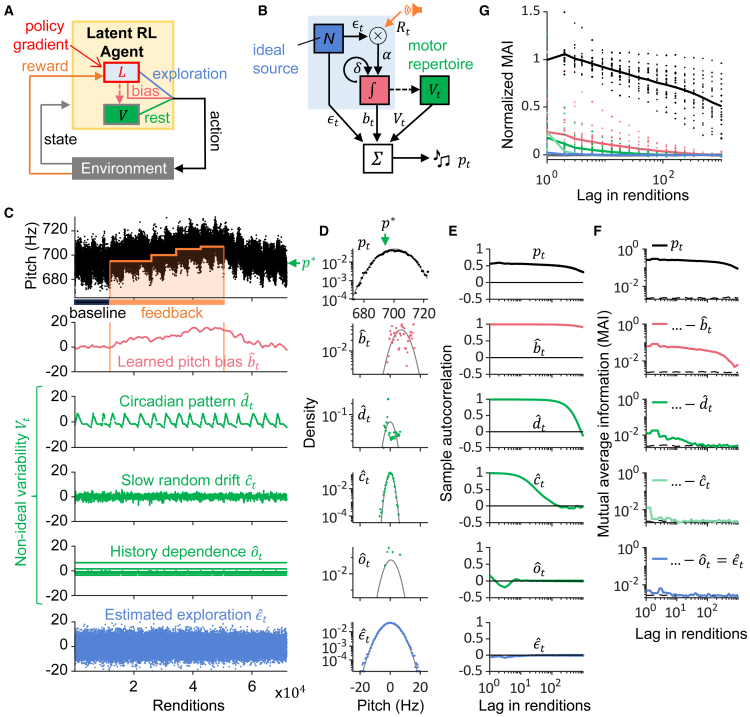
Latent RL self-consistently fits diverse learning trajectories (A) In latent RL, policy gradient search is restricted to a latent learner *L* that generates ideal explorations to learn a reward-increasing motor bias that may be consolidated into the motor repertoire *V* (the repertoire, however, is not directly influenced by reward). (B) We simulate a latent learner that produces explorations *ϵ*_*t*_ to increase reward *R*_*t*_ via a motor bias *b*_*t*_ that shifts the pitch *p*_*t*_ away from its motor repertoire *V*_*t*_ (green). The dashed arrow indicates motor consolidation that is not modeled. (C) After model fitting, the observed pitches *p*_*t*_ (black dots, top) are additively decomposed into their estimated components: the bias ${\hat{b}}_{t}$ (red), the motor repertoire ${\hat{V}}_{t}$ (green) composed of a target pitch *p*^∗^, a set of 3 fluctuations (a circadian pattern ${\hat{d}}_{t}$, a slow random drift ${\hat{c}}_{t}$, and a history dependence ${\hat{o}}_{t}$), and the exploration ${\hat{\epsilon }}_{t}$ (blue) with distributions shown in (D) and sample autocorrelations in (E). (F) The mutual average information (MAI) of the residuals formed by cumulatively subtracting from the pitches *p*_*t*_ (top) the estimated behavioral components. The MAI of the ultimate residual (blue, bottom) corresponding to the estimated explorations ${\hat{\epsilon }}_{t}$ is commensurate with sampled white noise (dashed). Same bird as in C-E. (G) Same as F but normalized and shown as averages (lines) across n = 18 birds (dots represent individual birds).

In latent RL, we assume there is a latent brain module that generates iid motor explorations *ϵ* and learns from them a motor bias *b* that tends to increase reward. Because these explorations are invisible to an outside observer, we refer to the learning process as “latent.” We further assume only the bias *b* is updated by reward, all other motor components *V* the agent can generate (the rest) are blind to reward (Figure 2A), which introduces non-optimality.

In the context of songbird pitch adaptation, the latent learner (Figure 2B):

1. Generates Gaussian iid trial-by-trial pitch explorations $\epsilon_{t}∼N\left({0,\sigma }_{\epsilon }^{2}\right)$;
2. It correlates the explorations with reward $R_{t-1}$ via a learning rate *α*, i.e., the presence/absence of WN, $\alpha R_{t-1}{\cdot \epsilon }_{t-1}$;
3. And it leakily integrates the correlation into a pitch bias $b_{t}={\left(1-\delta \right)b}_{t-1}+\alpha R_{t-1}\cdot \epsilon_{t-1}$. The leakiness *δ* models the attractive pitch target (the stable learned song) in the absence of WN. As absolute pitch bias increases, the leak term grows and increasingly counteracts feedback-driven changes. Thus, learning saturates when these forces balance, as seen in Figures 1C and 1D.

There is broad experimental support for computations 1–3 in the songbird anterior forebrain pathway (AFP), an analog cortico-basal ganglia pathway, that includes the dopaminergic recipient Area X in the basal ganglia and premotor output nucleus LMAN.

1. In support of ideal explorations (1), suppressing LMAN and Area X activity in both juveniles and adults greatly reduces the trial-by-trial variation of song.^29^^,^^39^^,^^40^ Also, LMAN neurons show delayed mirror responses, which arise under Hebbian learning only when they generate random sound features.^41^
2. In support of correlation (2), dopaminergic input feeding into the AFP is required for pitch conditioning,^34^ and the contingency between dopamine release and motor output sets the direction of the learned pitch bias.^42^^,^^43^
3. In support of integration (3), after lesions in the AFP, adult birds lose the ability to adapt pitch^32^; and inactivation studies reveal that LMAN biases song pitch to avoid penalties.^25^^,^^44^

The latent learner is characterized by mainly 3 parameters that we seek to infer from observed pitch trajectories: the (exploration) variance ${\sigma_{\epsilon }}^{2}$ of its iid explorations, the learning rate *α*, and the leakiness *δ* of the bias integrator.

We model the pitch *p*_*t*_ of syllable rendition *t* as a simple sum, $p_{t}=\epsilon_{t}+b_{t}+V_{t}$, where $\epsilon_{t}+b_{t}$ is the contribution of the learner, and *V*_*t*_ models all other components of a bird’s behavioral repertoire. We fit the parameters of *V*_*t*_ to baseline data and then kept them fixed during the feedback period (see STAR Methods “the model written as a kalman filter”). The model is successful when it achieves good fits to behavior and does so self-consistently, i.e., when the explorations estimated from data are close to ideal. This latter property can be tested via mutual information, a quantity that is minimized by independent (ideal) random variables. The deliberately non-optimal nature of this model is evident from the behavioral contribution *V*_*t*_, which is not assumed to depend on reward *R*_*t*_, only the bias *b*_*t*_ depends on reward (Figure 2A).

We found that four “other” behavioral components capture birds’ repertoire and make the latent learner self-consistent: $V_{t}={p^{*}+c}_{t}+d_{t}+o_{t}\text{,}$ where

1. *p*^∗^ is the stable pitch target,
2. *d*_*t*_ is a circadian pitch pattern,
3. *c*_*t*_ is a slow random drift, and
4. *o*_*t*_ models history dependence.

The circadian pattern *d*_*t*_ we modeled as a piecewise linear function (Figure S1A), the slow drift *c*_*t*_ as non-iid colored noise, and the pitch history dependence *o*_*t*_ as a function of the number of target syllables produced within the last 2 s (Figures S1B and S1C), for mathematical definitions see STAR Methods “latent reinforcement learning equations.” The estimated model components (Figure 2C) each contributed considerably to the total pitch autocorrelation (Figure 2E). Model self-consistency was evidenced by the estimated explorations being Gaussian distributed (Figure 2D) with flat autocorrelation (Figure 2E) and by the roughly iid statistics of explorations, evidenced by the small average lag-dependent mutual average information (MAI) (Figures 2F and 2G). Such self-consistency is not built into our model, which we demonstrate in simulations of hypothetical birds with non-ideal exploratory statistics (either non-Gaussian in Figure S2A or non iid in Figure S2B).

### A simpler classical reinforcement learning model is not self-consistent and fails to generate realistic pitch trajectories

**Figure 3:**
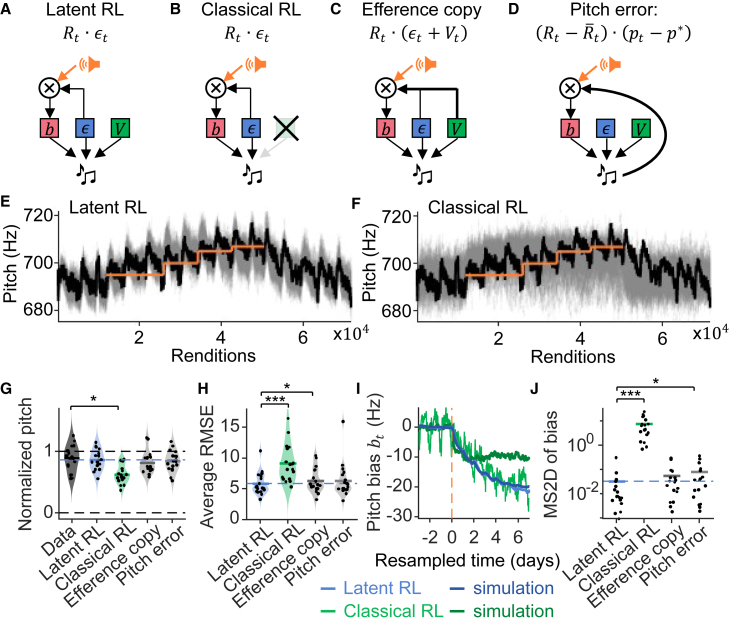
Latent RL provides better estimates of the learned pitch bias than does classical RL Illustration of model variants: (A) latent RL, (B) simple classical RL model without non-ideal fluctuations (setting $V_{t}=p^{*}$), and latent RL variants with more complex bias dynamics as in (C) the “efference copy” model $R_{t}\cdot \left(\epsilon_{t}+V_{t}\right)$ and (D) the “pitch error” model with $\left(R_{t}-{\bar{R}}_{t}\right)\cdot \left(p_{t}-p^{*}\right)\text{.}$ (E) The observed smoothed pitch trajectory (black line) lies within 100 smoothed trajectories of simulated latent RL (gray). Orange line depicts the pitch threshold. (F) Same as E, but for the classical RL model. The smoothed simulated trajectories (gray) change too quickly initially and do not reach sufficiently high pitches. (G) Normalized average pitch on the last feedback day, comparing data and fitted models. Normalization is such that the threshold on the first feedback day is 0 and on the last day 1 (dashed lines). All models agree with the data except classical RL (green line), which undershoots. (H) The RMSE averaged over all simulated trajectories for the latent RL (blue), classical RL (green), and the other two tested model variants (gray). (I) For an example bird, the pitch bias ${\hat{b}}_{t}$ (light blue) estimated with latent RL is a smooth function and similar to the average simulation (dark blue), whereas the pitch bias estimated with classical RL (light green) fluctuates too much (unlike in simulation, dark green). Day zero marks the beginning of the WN feedback. (J) The mean squared second derivative (MS2D) of the estimated pitch bias ${\hat{b}}_{t}$ is significantly lower (smoother) for latent RL than for classical RL or the pitch error model. Individual dots represent individual birds. ∗*p* < 0.05, ∗∗∗*p* < 0.001 by paired *t* test, n = 18 birds (G, H and J).

We found that the simpler “classical” RL model in which we leave out the fluctuating components (setting $V_{t}=p^{*}$) produced poor fits. Namely, the model $p_{t}=p^{*}+\epsilon_{t}+b_{t}$ (Figure 3B) encapsulates an optimal policy-gradient strategy in which the reward *R*_*t*_ is assumed to monotonically increase (see STAR Methods “alternative models”). The estimated explorations ${\hat{\epsilon }}_{t}$ (“estimation” is indicated by ˄) of this model displayed a MAI at lag 1 that was on average 2.5 ± 2.2 higher than the estimated explorations of the latent RL model (*p* = 0.03, *df* = 17, tstat = 2.4, two-tailed paired *t* test), thus less ideal than predicted by latent RL.

Latent RL generated pitch trajectories that closely resembled the observed behavior of birds (Figure 3E), but classical RL did not (Figure 3F), see STAR Methods “generative model.” Under latent RL, the normalized pitch values (normalized between 0 = the threshold on the first day of feedback and 1 = the threshold on the last day of feedback) of simulated data (with best-fit parameters for each bird) and of observed pitch values were indistinguishable on average on the last feedback day (observed 0.89 ± 0.21 vs. simulated 0.86 ± 0.16, *p* = 0.38, *df* = 17, tstat = 0.9, two-tailed paired *t* test, Figures 3A, 3E, 3G, and S3A), whereas the classical RL simulations underestimated the total learned pitch change (normalized pitch 0.63 ± 0.15, *p* = 2.4 · 10^−6^, *df* = 17, tstat = 6.93, two-tailed paired *t* test, Figures 3B, 3F, 3G, and S3B). Furthermore, after smoothing, the average root mean squared error (RMSE) between the simulated and observed pitch trajectories was much larger for classical RL than for latent RL (5.8 ± 1.8 Hz vs. 9.1 ± 3.0 Hz, *p* = 9.8 · 10^−8^, *df* = 17, tstat = 8.8, two-tailed paired *t* test, Figure 3H). Furthermore, latent RL produced smooth bias trajectories ${\hat{b}}_{t}$ (mean squared second derivative (MS2D) 0.03 ± 0.07), but classical RL produced bias trajectories that were on average 1300 times less smooth (7.6 ± 6.9, *p* = 0.0002, *df* = 17, tstat = −4.6, two-tailed paired *t* test, Figures 3I and 3J), implying that latent RL produces a learned bias that approximates well a policy gradient strategy, whereas classical RL does not.

We further tested two alternative models in agreement with the latent RL assumptions: First, we tested an “efference copy” model (Figure 3C) in which the bias changes according to $R_{t}\cdot \left(\epsilon_{t}+V_{t}\right)\text{,}$ i.e., the AFP receives an efference copy of the entire, summed behavioral repertoire. Second, we tested a “pitch error” model (Figure 3D) in which the bias changes according to $\left(R_{t}-{\bar{R}}_{t}\right)\cdot \left(p_{t}-p^{*}\right)\text{,}$ where $p_{t}-p^{*}$ is the pitch error and ${\bar{R}}_{t}$ is the mean reward over that last 20 renditions (its subtraction ensures that one of the two multiplicands has mean zero).

These two latent RL model variants did not improve the performance of the basic latent RL model defined by $R_{t}\cdot \epsilon_{t}$. Compared to the basic latent RL model, the MAI of the estimated explorations ${\hat{\epsilon }}_{t}$ at lag 1 was larger on average both for the “efference copy” model (1.1 ± 0.2 times larger, *p* = 0.05, *df* = 17, tstat = 2.1, same test) and for the “pitch error” model (1.1 ± 0.3 times larger, *p* = 0.11, *df* = 17, tstat = 1.7). Their estimated pitch biases tended to be less smooth (“efference copy”: 0.06 ± 0.09, 3.5 times higher than basic latent RL, *p* = 0.14, *df* = 17, tstat = −1.6, “pitch error”: 0.08 ± 0.10, 4.2 times higher, *p* = 0.03, *df* = 17, tstat = −2.4, Figure 3J), suggesting they form worse approximations of a policy gradient strategy. The models tended to slightly undershoot the total observed pitch change on the last feedback day on average (0.82 ± 0.18 normalized pitch, *p* = 0.10, *df* = 17, tstat = 1.72, “efference copy,” 0.84 ± 0.17 normalized pitch, *p* = 0.20, *df* = 17, tstat = 1.34, “pitch error,” Figure 3G) and they produced a higher average RMSE than did basic latent RL (6.3 ± 2.0 Hz, *p* = 0.04, *df* = 17, tstat = 2.2 “efference copy,” 6.3 ± 2.8 Hz, *p* = 0.08, *df* = 17, tstat = 1.9, “pitch error,” Figure 3H). Thus, although both these model variants are more complex than basic latent RL, neither significantly improved the fits nor did they display higher self-consistency.

### Estimating the motor exploration

**Figure 4:**
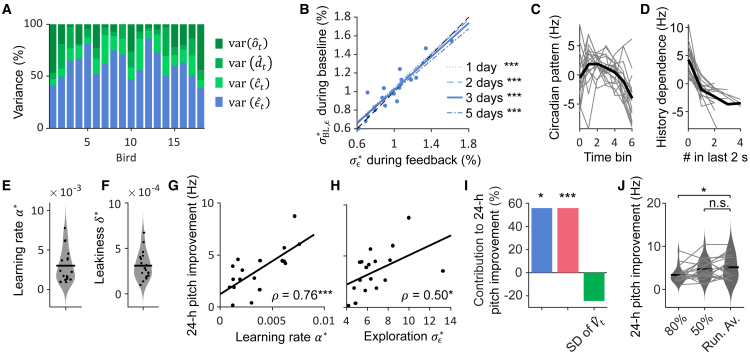
Best-fit learning parameters (A) The estimated contribution of exploration (blue) to the overall variability in pitch ranges from 41 to 91% for different birds (bars). The other behavioral components — colored noise (light green), circadian pattern (green), and history dependence (dark green) — make up the rest of the variability. (B) The best-fit standard deviation of exploration $\sigma_{\epsilon }^{*}$ in percent of *p*^∗^ generated by the latent learner (blue dots, n = 18 birds) during the feedback period (x axis) and during baseline (y axis). Linear fits (blue lines, ∗∗∗ represents *p* < 0.001) for different numbers of baseline days used (legend), the fits are close to the diagonal (black dashed line). (C) The average circadian pattern (black) increases in the morning and then decreases over the course of the day (individual birds shown in gray). (D) The average history dependence (black) decreases with increasing number of renditions within the last 2 s (individual birds shown in gray). (E and F) Distribution of learning rates *α*^∗^ (E) and leakiness *δ*^∗^ (F). Dots represent individual birds, and the line indicates the mean. (G and H) The learning rate *α*^∗^ (G) and the exploration standard deviation $\sigma_{\epsilon }^{*}$ (H) are significantly correlated with daily pitch improvement during the first three feedback days (Pearson correlation coefficient, ∗*p* < 0.05, ∗∗∗*p* < 0.001, n = 18 birds). (I) The percentage estimated contribution to pitch the improvement of the various fixed effects of a linear mixed effects model (∗*p* < 0.05, ∗∗∗*p* < 0.001) confirmed a positive effect of exploration and learning rate. (J) Learned pitch of simulated birds (individual lines) increases slowly when updating the threshold daily to the 80^th^ percentile (80%) and more rapidly when they are updated either to the median (50%) of the previous day or continuously to the median of the last 20 renditions (Run. Av.), ∗*p* < 0.05 by paired *t* test, n = 18 birds.

Latent RL is designed to tease out the behavioral component that serves as motor exploration. We expressed the estimated variance ${\sigma_{\epsilon }^{*}}^{2}$ of exploration (“best fit” is indicated by ∗) during WN as a fraction $f={\sigma_{\epsilon }^{*}}^{2}/{\hat{\sigma }}_{BL\;}^{2}$ of the total baseline (BL) pitch variance ${\hat{\sigma }}_{BL}^{2}=\text{var}\left(p_{t}\right)$ directly estimated from the pitch measurements. We found an average exploration fraction *f* of 61 ± 14% (*n* = 18 birds, Figure 4A); the broad range of *f* from 41 to 91% suggests that animals differed in their propensity to explore over a factor of more than two (or, put differently, they differed by a factor of two in their proportion of non-explorative behavioral components).

We also estimated explorations during the baseline period, when the pitch dynamics were similarly complex as during the feedback period. The standard deviation $\sigma_{\text{BL},\epsilon }^{*}$ of exploration divided by the target pitch was highly correlated with $\sigma_{\epsilon }^{*}$ computed during the feedback period (Pearson correlation coefficient *ρ* = 0.91, *p* = 1.1 · 10^−7^, n = 18 birds, see STAR Methods “exploration variance,” Figure 4B). The slope *m* of a linear fit of $\sigma_{\text{BL},\epsilon }^{*}$ against $\sigma_{\epsilon }^{*}$ was not different from the unit slope (*m* = 1.02, *p* = 0.5, n = 18 birds, *t* test), suggesting that birds do not modulate their exploration variance during song practice, even while experiencing aversive stimuli.

Explorations and their standard deviation (SD) could be reliably estimated from few renditions only. The estimated baseline exploration $\sigma_{\text{BL},\epsilon }^{*}$ from half a day of song data was highly correlated with $\sigma_{\epsilon }^{*}$ at *ρ* = 0.87 (*p* = 2.5 · 10^−6^, n = 18 birds). When estimated from merely 100 consecutive syllable renditions, the baseline exploration was still correlated at *ρ* = 0.76 (*p* = 2.2 · 10^−4^, n = 18 birds) and from 20 consecutive renditions it was correlated at *ρ* = 0.89 (*p* = 5.8 · 10^−7^, n = 18 birds). That the exploration SD $\sigma_{\epsilon }^{*}$ can be estimated from only 20 renditions, suggesting that the momentary pitch variability (the standard deviation of pitch across 20 renditions) is a good proxy of the estimated amount of exploration. Indeed, the average momentary variability was correlated with $\sigma_{\epsilon }^{*}$ at *ρ* = 0.89 (*p* = 7.6 · 10^−7^, n = 18 birds, SD = 1.1 ± 0.2% of *p*^∗^). Within individual birds, the momentary variability was quite noisy, however, showing an average standard deviation of 18 ± 2% (mean ± SD, m = 1000 sets of 20 pitches in each of n = 18 birds).

### Estimated motor fluctuations and model parameters

The estimated motor fluctuations part of ${\hat{V}}_{t}$ (their variances) were on average roughly evenly split between the colored noise (14 ± 7%), the circadian pattern (11 ± 8%), and the history dependence (15 ± 14%, n = 18 birds, Figure 4A). The average circadian pattern followed an inverted U-shape with overall lower pitch values toward the end of the day (decreased over the day: 3.1 ± 6.1 Hz, *p* = 0.04, *df* = 17, tstat = −2.18, two-tailed paired *t* test, Figure 4C). There was a significant average pitch decrease from the first motif in a song bout (0 renditions within the last 2 s) to the second motif (5.3 ± 3.1 Hz, *p* = 1.3 · 10^−6^, *df* = 17, tstat = −7.29, two-tailed paired *t* test, Figure 4D).

The learning rate *α*^∗^ was on average 0.003 ± 0.002 (range 0.001–0.008, Figure 4E). This large range suggests that some birds are fast and others are slow learners. The average leakiness *δ*^∗^ was 0.0003 ± 0.0002 (range 0.0001–0.0007, Figure 4F), suggesting a similarly large spread of pitch recovery rates after the feedback period.

### Effects on daily pitch improvement

The learning rate *α*^∗^ was significantly correlated with the feedback-induced daily pitch improvement measured over the course of 24-h periods (Pearson correlation coefficient *ρ* = 0.76, *p* = 0.0003, n = 18 birds, Figure 4G). There was also a significant correlation between the daily pitch improvement and the exploration SD $\sigma_{\epsilon }^{*}$ (Pearson correlation coefficient *ρ* = 0.50, *p* = 0.04, n = 18 birds, Figure 4H) but not between the pitch improvement and the standard deviation of estimated motor fluctuations ${\hat{V}}_{t}$ (Pearson correlation coefficient *ρ* = 0.06, *p* = 0.80, n = 18 birds). We confirmed this using a linear mixed effect model of the daily pitch improvement with fixed effects given by the exploration SD $\sigma_{\epsilon }^{*}$, the learning rate *α*^∗^, and the SD of ${\hat{V}}_{t}$ (see STAR Methods “contribution to learning”). The model confirmed a significant positive effect of exploration (fixed effect = 0.3, *p* = 0.04) and the learning rate (fixed effect = 661, *p* = 0.0002), and a non-significant negative effect of motor fluctuations ${\hat{V}}_{t}$ (fixed effect = −0.1, *p* = 0.63), Figure 4I. In line with the model’s prediction, we did not find evidence that the direction of the circadian pattern relative to the direction of experimentally driven pitch changes influences learning. Birds with circadian patterns aligned with or opposed to the learning direction shifted their pitch by similar absolute daily amounts on average (3.5 ± 2.9 Hz vs. 3.6 ± 1.3 Hz, *p* = 0.94, *df* = 16, tstat = −0.08, two-sided two sampled *t* test).

### Optimal training paradigm

Latent RL predicts that the learned daily pitch change should be maximal when explorations sample WN stimuli with probability ½, corresponding to the maximum information gain (see Supplement: Theoretical learning speeds and limits). In simulated experiments (with best fit parameters for each bird), the daily pitch improvement was 5.1 ± 2.8 Hz when the threshold continuously tracked the median of the last 20 renditions, Figure 4J. The daily pitch improvement was about the same when the threshold was set each day to the median of the previous day’s pitch distribution (4.7 ± 2.4 Hz, *p* = 0.49, tstat = −0.70, n = 18 simulated birds, two-tailed paired *t* test) but it was significantly smaller when the threshold was set to the 80^th^ pitch percentile of the previous day’s distribution (3.4 ± 1.9 Hz, *p* = 0.02, tstat = −2.63, n = 18 simulated birds, two-tailed paired *t* test, Figure 4J). In these simulated experiments, the learning rate *α*^∗^ did not depend on the threshold update strategy (Figure S4), showing robustness of the best-fit learning rate to experimental details.

### The exploration variance predicts the effects of brain lesions

**Figure 5:**
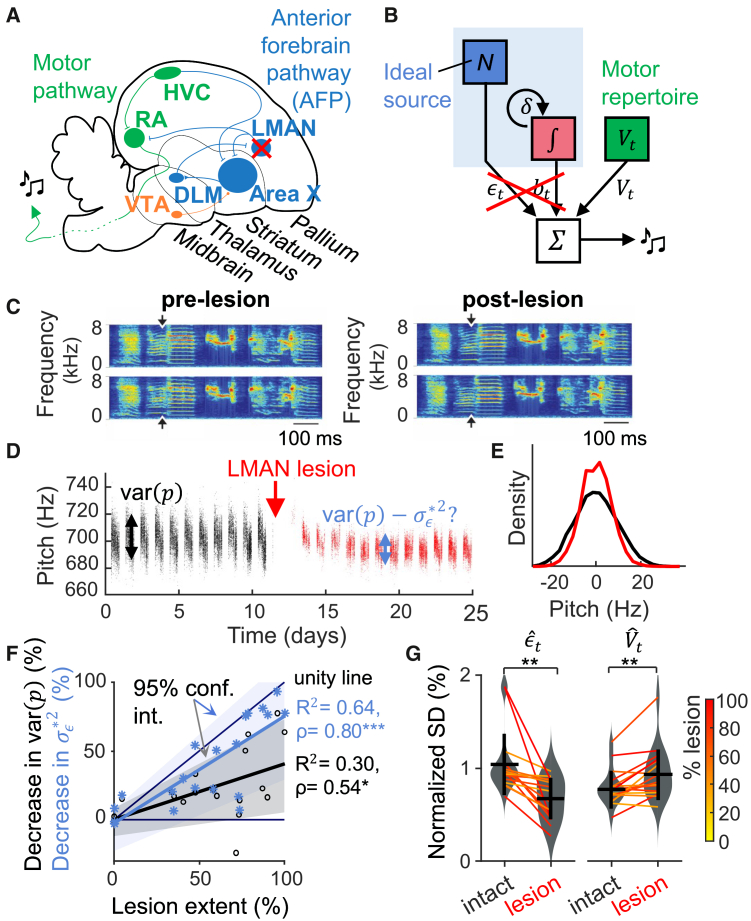
LMAN lesions exclusively eliminate explorations (A) Brain schematic of the song system. We bilaterally lesioned the output nucleus of the AFP, the lateral magnocellular nucleus of the anterior nidopallium (LMAN). (B) We hypothesized that LMAN lesions abolish both explorations *ϵ*_*t*_ and the learned pitch bias *b*_*t*_. (C) Two example song motifs before (left) and after (right) LMAN lesions. The lesions did not degrade the spectro-temporal motif structure. The arrows indicate the measurement window of the target syllable’s pitch. (D) Pitch trajectory of an example bird showing reduced pitch variability (red) following LMAN lesion. (E) Normalized histogram (density) of mean-subtracted pitch before (black) and after (red) LMAN lesion (same bird as in D). (F) The decrease in exploration variance ${\sigma_{\epsilon }^{*}}^{2}$ (blue) correlates better with lesion extent than does the decrease in pitch variance (black), n = 20 birds. Shown is the type-1 linear regression and 95% confidence interval estimated from 100 bootstrapped replicates. The reported statistics are the Pearson correlation coefficient ρ and the explained variance *R*^2^, ∗*p* < 0.05, and ∗∗∗*p* < 0.001. (G) Change in exploration standard deviation (SD) normalized to the target pitch before and after LMAN lesion (left) and change in normalized SD of remaining behavioral components *V*_*t*_ (right), n = 17 birds (thin lines) with non-zero lesion volume and their average and SD (black line and error bar), ∗∗*p* < 0.01 by paired *t* test.

We further tested latent RL as a plausible neural mechanism, i.e., whether the learner module captures the role of a motor-learning pathway. Pitch adaptation in songbirds depends on a cortico-basal ganglia loop: Without its output nucleus (LMAN), song variability is largely reduced^23^ and adult birds fail to learn from aversive pitch reinforcement.^26^ We thus hypothesized that the explorations match the pitch variance contributed by LMAN. Accordingly, it should be possible to simulate the effects of bilateral lesions in LMAN (Figure 5A) by fitting a latent RL model to baseline pre-lesion data and then to consider the exploration variance as the predicted reduction in pitch variance caused by the lesions (Figure 5B). This model should predict lesion effects on song better than a simpler model that assumes all pitch variance is generated by LMAN (including the fluctuations) analog to the classical RL model.

In line with previous reports,^39^^,^^45^^,^^46^ lesions did not affect the overall spectral and temporal structure of song motifs (example in Figure 5C), but they decreased the average daily pitch variance by 22 ± 25% on average (n = 20 birds, Figures 5D–5F). Across birds, lesion extent was moderately correlated with the reduction in observed pitch variance (Pearson correlation *ρ* = 0.54, *p* = 0.01, n = 20 birds, regression fit explains 30% of variance, Figures 5F and S5). In contrast, lesions reduced the best-fit exploration variance ${\sigma_{\epsilon }^{*}}^{2}$ by 40 ± 32% on average and lesion extent was strongly correlated with exploration decrease (ρ = 0.80, *p* = 2.1 · 10^−5^, regression fit explained 64% of the variance, Figure 5F). Thus, exploration was a better predictor of LMAN lesion outcome than overall pitch variability. We found that exploration was also a better predictor of lesion outcome than a simpler model^47^ in which LMAN’s effect is estimated as the motor residual following pitch detrending (ρ = 0.67, *p* = 0.001, n = 20 birds, regression fit explained 45% of the variance). Thus, neither pitch nor detrended pitch captures LMAN function as well as does pitch exploration, in support of our hypothesis that LMAN generates ideal pitch explorations.

Our model further predicts that only exploration ${\hat{\epsilon }}_{t}$ should decrease after LMAN lesion, but not the remaining behavioral components ${\hat{V}}_{t}$ that are not part of the latent learner. Indeed, the standard deviation of the exploration normalized by the target pitch decreased by 32 ± 24% (from 1.0 ± 0.33, to 0.68 ± 0.22, *p* = 0.001, n = 17 lesioned birds with non-zero lesion volume, tstat = −4.03, two-tailed paired *t* test) whereas the standard deviation of non-ideal sources of variability even increased slightly by 22 ± 24% (from 0.78 ± 0.20% to 0.94 ± 0.27%, *p* = 0.002, n = 17 lesioned birds with non-zero lesion volume, tstat = 3.81, two-tailed paired *t* test), Figure 5G. Importantly, whereas the decrease in exploration correlated with lesion size (Figure 5F), the increase in ${\hat{V}}_{t}$ did not (*ρ* = 0.26, *p* = 0.26), suggesting that the change in motor fluctuations might be a general effect of surgery instead of being specific to lesioning LMAN. Specifically, there is no change in circadian pattern after LMAN lesions (−0.03 ± 0.18%, *p* = 0.54, tstat = −0.63, n = 17, two-tailed paired *t* test). The lesion study supports our modular assumption that LMAN generates iid motor explorations but not non-ideal motor fluctuations.

### Human reinforcement learning of pitch

**Figure 6:**
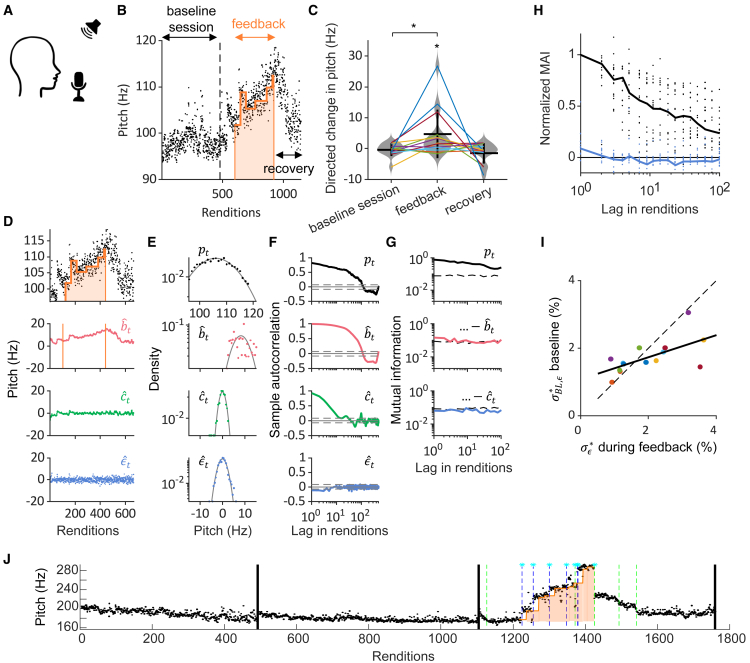
Pitch conditioning in human subjects (A) Subjects were instructed to repeat the utterance “da” at a rate of roughly twice per second. Same pitch paradigm as in birds. (B) Pitches (dots) of “da” before, during, and after contingent WN feedback, in an example subject. During the baseline session, no WN is played. During minutes 2–5 of the feedback session (delimited by the orange curve), aversive WN stimuli are played upon low pitch renditions. During recovery, no WN is played. (C) Pitch change (away from the WN zone, e.g., up in B) during the baseline session, during the feedback period, and during recovery (n = 14 subjects indicated by lines, ∗*p* < 0.05 by paired *t* test). (D) After model fitting, the pitches *p*_*t*_ (black, top, same subject as in b) are additively decomposed into a bias ${\hat{b}}_{t}$ (red), a colored noise ${\hat{c}}_{t}$ (green), and the estimated exploration ${\hat{\epsilon }}_{t}$ (blue) with distributions shown in (E) and sample autocorrelation in (F). (G) The mutual average information (MAI) of the residuals (top to bottom, formed by cumulatively subtracting the indicated behavioral components) from the pitches *p*_*t*_ are gradually reduced. The MAI of the estimated explorations ${\hat{\epsilon }}_{t}$ (blue, bottom) is as low as that of sampled white noise (dashed line). (H) The normalized MAI of pitch trajectories (black dots, n = 14 subjects) and of estimated explorations (blue dots, n = 14 subjects), and their averages (lines). (I) The exploration standard deviation $\sigma_{\epsilon }^{*}$ in percent of *p*^∗^ (colored dots, n = 14 subjects) is significantly correlated between baseline (y axis) and feedback (x axis) periods (linear fit: solid line, *p* = 0.001, Pearson’s correlation; identity: dashed line). (J) Subject who increases the pitch step rate in the direction of WN escapes (6 of 8 steps during WN are aligned with the WN escape direction, *Z* = 2.0). Pitch steps preceded by WN are indicated by a cyan asterisk, up steps by dashed blue lines, down steps by dashed green lines).

We found that our approach also holds promise in human subjects that were instructed to repeat a simple utterance while trying to avoid WN stimuli that we delivered contingently on their pitch (see STAR Methods “pitch conditioning in humans”). During WN sessions, subjects successfully changed the pitch away from the WN zone, and after the cessation of WN, they tended to revert their pitch back toward baseline (Figures 6A–6C). A simple latent RL model that included merely an additional colored noise source (and target pitch) as a non-ideal source of variability proved sufficient to self-consistently estimate subjects’ pitch explorations (Figures 6D–6H). The standard deviations $\sigma_{\epsilon }^{*}$ during the feedback period and $\sigma_{\text{BL},\epsilon }^{*}$ during baseline, both normalized to *p*^∗^, were correlated (correlation coefficient *ρ* = 0.63, *p* = 0.017, n = 14 human subjects, Figure 6I). However, the slope *m* = 0.78 of a linear fit of the feedback vs. baseline variance was different from unity (*m* = 1.0, *p* = 0.01, *t* test, n = 14), suggesting that subjects increased the exploration variance during the feedback period (as in active inference).

Humans differ from zebra finches in that the former can dramatically change vocal pitch from one rendition of an utterance to the next,^48^ a possible expression of stepwise exploration or insight learning (a form of exploitation). Indeed, in zebra finches, we found no pitch discontinuities or steps where past and future pitch averages would differ by more than Θ = 5% (see STAR Methods “pitch steps,” Figure S6). However, in 14 human subjects we found a total of 103 pitch steps (Θ = 5%, mean ± SD: 3.6 ± 4.6 steps per subject, range 0–14 steps).

Pitch steps could constitute a human exploratory strategy on their own, since among the 103 pitch steps, 51 (50%) were preceded by recent WN experience (in the last 20 utterances), many more than the chance level of 20% defined as the fraction of utterances with preceding WN (test for equal proportions, *p* < 10^−24^, see STAR Methods “pitch steps”). Five of fourteen subjects significantly changed their pitch stepping behavior in response to WN. All five subjects produced significantly more pitch steps during WN than during baseline (test of equal proportions, *p* < 0.05), suggesting that they adopted a pitch stepping strategy to explore the contingency of WN penalties.

Individual subjects produced too few pitch steps during WN (mean 3.6, range 0–14 steps) to test whether they learned also to produce pitch steps as an exploitative strategy, with the exception of one subject who seemingly used pitch steps to escape WN (6/8 steps during WN were directed toward escape, *Z* = 2.0, Figure 6J). Thus, although not all subjects seemed to make use of pitch stepping, some seemed to use it as a directed vocal exploration strategy, and one managed to skillfully use stepping to escape WN. When eliminating the discontinuities (see STAR Methods “pitch steps”), the correlation between exploration variance during baseline and the feedback period remained high (*ρ* = 0.71, *p* = 0.005, *m* = 0.77). Robust correlations were observed for a wide range of thresholds (Θ = 1%, 10%), suggesting that subjects consistently differed in their magnitude of exploration, despite the aversive stimulus and despite a possible pitch-stepping strategy.

## Discussion

To overcome the misalignment between RL theory and animals’ action choices, we have introduced a latent learner that agrees with a wealth of published data and that produces excellent fits to behavior even during sub-optimal (non-gradient-like) learning periods. By defining explorations as ideal motor perturbations, we could estimate their impact on behavior on a trial-by-trial basis, both in task and task-free settings.

Latent RL abandons unrealistic notions that behavior must be either optimal or explorative, as assumed by classical exploitation-exploration tradeoffs. By restricting exploration to a learner’s ideal perturbations, we allocate optimal (gradient-following) learning to a distinct behavioral module rather than to the entire motor repertoire. This restricted latent optimality of the latent module makes latent RL a normative theory, providing a framework for disentangling the components of a motor gesture, in particular, the component that subjects use to learn motor skills.

Our work suggests that birdsong learning is driven by an active Gaussian exploratory process, contradicting earlier work that proposed learning to be driven by non-Gaussian pitch statistics.^49^ The disagreement with our work likely stems from the non-discriminatory summing in their model across diverse syllable types and processes, both of which can lead to long-tailed pitch statistics. By contrast, we tried to estimate the latent components of single syllables, showing that self-consistent behavior decomposition is possible from ideal Gaussian exploration. In hindsight, because the pitch variance from as few as 20 renditions forms a good estimate of exploration variance, previous studies analyzing little song data^39^^,^^46^^,^^50^ were, unknowingly, studying a good proxy of exploration as estimated with latent RL.

Our work supports the validity of first de-trending pitch trajectories from circadian patterns before analyzing pitch dynamics, since these trends are well accounted for by a fixed additive component. Moreover, our work suggests that to quantify learning in pitch conditioning paradigms, especially when comparing between studies with different threshold update strategies,^26^^,^^31^^,^^33^ the learning rate *α* may provide a more consistent and interpretable measure of learning performance than daily pitch shifts, since *α* remains unaffected by the particular update strategy used (Figures 4 and S4).

Our analysis provides evidence that birds learn on a trial-by-trial basis from the explorations generated by the latent learner, but we find no evidence for overnight learning (Figure 1). Our findings thus align with recent work that similarly ruled out sleep-related overnight learning in adult songbirds^51^ and in juveniles during developmental song learning.^52^ The overnight discontinuities we find appear to be inflexible expressions of circadian patterns. Since such patterns are part of every cell’s life cycle and are evolutionary much older than vertebrates and their motor exploration strategies, we speculate that circadian patterns of behavior might derive from a collective cellular need that is more important than the hypothetical advantage of maximizing reward.

Although circadian patterns appear to introduce useless variability, birds might be able to exploit this variability thanks to its widening effect of the search horizon. Namely, the self-reinforcing intrinsic reward during developmental song learning is a binary-like function of difference between the current pitch and a sensory target.^47^ Circadian pitch patterns might bring a syllable close enough to the target to trigger intrinsic reward from which the target can be learned. The idea is comparable to imprecisely digging a tunnel: variability in digging sites might lead to the discovery of softer soil, making it possible to break through challenging structures. By clarifying how different sources of variability contribute to learning, latent RL provides a more nuanced characterization of cortico-basal ganglia function than previous models, in which all motor variability was interpreted as exploration,^47^^,^^53^ including models that refer to exploration as noise.^54^

Latent RL differs from previous work in that it assumes that ideal exploration is not merely sufficient; it is necessary as a model assumption. What we gain from this assumption is the ability to estimate the motor exploration inherent in as little as a single motor rendition. For sufficiency of iid exploration, there is broad support, since iid (e.g., Gaussian white) motor variability is a standard component of models of behavioral learning.^18^^,^^19^^,^^22^^,^^55^^,^^56^^,^^57^^,^^58^ The iid assumption is usually motivated by convenience rather than as a criterion of self-consistency. Likely, assumed iid variability produces good fits because human motor adaptation is usually studied during short periods with few trials,^59^^,^^60^ in which sub-optimal learning trajectories might not necessarily show up (e.g., Figure 6). That ideal exploration is also necessary is less obvious, but is suggested by a recent study on the microstructure of learning in humans. Findling et al. attributed non-optimality of human behavior to a noise source that engendered behavioral correlations across successive decisions,^17^ i.e., a non-ideal source in our terms. Whereas these authors attributed behavioral non-optimality to computational noise, we attribute it more broadly to arbitrary behavioral modules, such as circadian fluctuations, that contribute non-ideal fluctuations and that are uninformed about trial-by-trial reward. We recognize that peripheral (e.g., muscle) and other unavoidable sources of behavioral noise may be approximately iid as well, but the presumed evolutionary efficiency led us to assume that their magnitude should be small (i.e., neglectable), especially during skilled behaviors where variability is inversely related to fitness.

Ideal statistics of exploration perfectly align with the brain’s sensitivity to sensory errors and its ability to modulate error sensitivity.^55^^,^^61^ Correlated errors increase sensitivity and speed up learning of compensatory responses, which might be relevant when pitch is persistently too high or too low.^62^ Self-generated perturbations of behavioral output will also cause fluctuations of sensory feedback (sensory errors). But when perturbations are ideal, consecutive fluctuations are uncorrelated in sign and so do not affect sensitivity to sensory errors.^55^ Thus, ideal perturbations by themselves do not trigger known mechanisms for adapting to external perturbations. The sensory apparatus thus seems to filter out self-generated sensory fluctuations with ideal statistics. Ideal experimentation therefore promotes adaptability in a dynamic world, perhaps in the service of robustly finding the true causes of increasing reward.

By sub-optimality of latent RL, the latent learner can deliberately dismiss some information, such as motor efference copies (Figure 3), because they are dispensable for generating pure randomness. As such, latent RL argues against the existence of motor efference copies of pitch variability in LMAN,^26^ in line with a more recent proposal whereby LMAN needs no efference copy provided it receives information about an auditory target which it can translate into a directional motor command, as is characteristic of inverse models.^48^

In this vein, we see latent RL as an evolutionary old adaptation that encapsulates unconscious motor learning that is cognitively effortless and merely requires practice, such as riding a bike. That does not mean that the learner’s output, in particular its bias, could not be influenced by evolutionarily younger mechanisms. For example, upstream cortical modules could influence the learned bias to give rise to directed exploration.^63^ Such directed exploration could, for example, take the form of pitch stepping. Future work would need to explore whether latent RL is permissive of such higher-level conscious learning mechanisms.

Past work suggests that exploration can also be modulated: Motor variability can depend on feedback,^59^ and it can be regulated by both fast and slow reward-dependent processes.^57^ In our experiment, zebra finches did not appear to modulate the magnitude of ideal exploration, but the assumed additive nature of explorations would make it simple to model that. Notably, zebra finches can recruit variability to direct learning to a subset of motor gestures,^64^ and they change variability with behavioral context, e.g., singing more stereotyped songs when directed to a female, which is accompanied by reduced bursting in LMAN neurons.^65^ It remains to be seen which of these modulations of variability can be ascribed to exploration. Also, extending our framework to juvenile song, which is intrinsically more variable, could help disentangle how much of motor variability reflects true exploration versus useless noise, and potentially reveal the modulation of exploration during development. Our reduced paradigm of singly housed birds seemingly did not elicit these modulations, but conceptually, latent RL can deal with such modulation as seen in humans (Figure 6I).

Latent RL holds promise as a simple strategy for building complex motor programs from simpler ones. In our model, latent RL merely provides a simple bias toward more rewarding behavioral variants, but the learner module is oblivious of how to meaningfully accumulate and consolidate the bias into extensive motor programs and motor plans. Unlike classical RL, the challenge in latent RL is to transfer the learned bias gained from self-perturbations to motor primitives and more complex programs that control behavior in non-random ways. In this sense, latent RL can be seen as part of a more general perturbator-consolidator architecture (rather than an actor-critic architecture). In songbirds, the learned bias generated by LMAN is consolidated in HVC (proper name) and the robust nucleus of the arcopallium.^51^ Processes of consolidation could be conceptualized in future work as a transfer of the learned bias to the repertoire of behavioral primitives to incorporate ideal exploration into more elaborate models of behavioral learning.

### Limitations of the study

While latent reinforcement learning provides a normative framework for decomposing behavioral variability, several limitations remain.

First, the non-ideal components we model (circadian pattern, slow random drift, and history dependence) are treated as additive statistical contributions. How to computationally identify these non-ideal components (their equations) is an open theoretical problem. Also, the biological origins of these components remain to be identified, raising the question of whether they map onto distinct neural processes or peripheral factors such as muscle properties. Identifying the biological origins would be important for better understanding how exploratory and non-exploratory variability are implemented in the brain and would allow a transition from the current phenomenological parametrization of non-ideal variability to a more mechanistically grounded formulation. For one, it would be interesting to find out whether circadian patterns exert a direct influence on the motor system or indirectly by biasing perception, which could be examined in combined singing and perceptual memorization tasks.

Second, it remains an open challenge to identify the mechanistic basis of ideal explorations. Diverse mechanisms have been proposed to generate randomness on multiple spatial scales, ranging from individual ion channels to spontaneous synaptic vesicle release to recurrent neural networks under inhibitory-excitatory balance.^66^^,^^67^^,^^68^ On top of that, ideal perturbations could be promoted by synaptic learning rules that act on mutual information,^69^^,^^70^ in line with the brain’s strategy to remove temporal correlations.^71^

Third, the framework assumes that ideal exploration follows Gaussian and independent statistics. We validated this assumption empirically (Figure S2), but it may not hold in other species or behavioral contexts where exploratory dynamics deviate from these distributions. For example, while the latent RL model describes a meaningful exploratory component in humans, it does not account for the full range of human learning strategies, even in the simple paradigm we studied. Human pitch stepping can be viewed as an extreme case of modulating exploration magnitude. On the one hand, large explorations allow for reaching more distant targets and rewards and so seem desirable—even though large explorations do not necessarily imply faster learning, since we find learning speed to depend more sensitively on the learning rate than on exploration magnitude, unlike in.^19^^,^^22^ On the other hand, large explorations may be a handicap, since the brain often learns more from small errors (perturbations) than from large errors.^33^^,^^72^^,^^73^^,^^74^^,^^75^ The pitch stepping we find in humans might thus be a mechanism to flexibly bypass the pitfalls of too-large ideal explorations. Pitch stepping harbors the obvious benefit of serving to explore more distant rewards in fewer attempts (steps), reminiscent of Levy flight foraging.^76^ By being flexibly engageable (i.e., during feedback periods), explorative stepping would be no obstacle to skilled stereotyped behavior, which needs further investigation.

Fourth, on the practical implementation side, currently, our Kalman model and parameter fitting via expectation maximization allow only for simple linear models. How to extend our work to nonlinear models is an important avenue for future work.

Addressing these limitations in future studies will help bridge latent RL’s statistical decomposition with its biological implementation and clarify how different sources of behavioral variability contribute to adaptive motor learning.

## Resource availability

### Lead contact

Requests for further information and resources should be directed to and will be fulfilled by the lead contact, Anja T. Zai (zaia@ethz.ch).

### Materials availability

This study did not generate new unique reagents.

### Data and code availability

• The raw pitch data have been deposited at ETH Research Collection and are publicly available as of the date of publication at DOI: https://doi.org/10.3929/ethz-c-000787082.
• All original code has been deposited at ETH Research Collection and is publicly available at https://doi.org/10.3929/ethz-c-000787082 as of the date of publication.
• Any additional information required to reanalyze the data reported in this article is available from the lead contact upon request.

## Acknowledgments

This work was supported by the 10.13039/501100001711Swiss National Science Foundation, Projects 31003A_182638 and 205320_215494/1, and Agreement 51NF40_180888. We thank Kristina Biedermann for helping to set up the human recordings and Mai Akahoshi for the illustration of the bird used in the graphical abstract.

## Author contributions

Conceptualization, A.T.Z. and R.H.R.H.; methodology, A.T.Z. and R.H.R.H.; formal analysis, A.T.Z. and R.H.R.H.; investigation, A.T.Z., C.L., and S.S.; data curation, A.T.Z., C.L., and S.S.; writing – original draft, A.T.Z. and R.H.R.H.; writing – review and editing, A.T.Z., C.L., N.G., and R.H.R.H.; supervision, N.G. and R.H.R.H.; authors have read and approved the final version, A.T.Z., C.L., S.S., N.G., and R.H.R.H.

## Declaration of interests

The authors declare no competing interests.

## STAR★Methods

### Key resources table

**Table:** 

REAGENT or RESOURCE	SOURCE	IDENTIFIER
**Deposited data**		

Raw pitch values	This Paper; ETH Research Collection	https://doi.org/10.3929/ethz-c-000787082

**Software and algorithms**		

Latent RL parameter estimation and code (MATLAB) to reproduce analysis presented in this paper.	This Paper; ETH Research Collection	https://doi.org/10.3929/ethz-c-000787082

### Experimental model and study participant details

#### Animal model

We used a total of 35 male zebra finches (Taeniopygia guttata) that were bred and raised in our animal facilities either in Zurich (Switzerland) or Orsay (France). The animals’ ages at the beginning of the experiment were in the range of 90-400 days post hatch (dph) except for one bird that was above 800 dph. During the experiment, birds were individually housed in sound-attenuating recording chambers with a 14/10-hour day/night cycle. We used a total of n = 20 birds for LMAN lesions, from which n = 8 birds were pitch reinforced prior to lesioning LMAN. We reanalyzed pitch-reinforcement data in n = 18 birds from.^48^ All experimental procedures were performed in accordance with the Veterinary Office of the Canton of Zurich (License number ZH123/2010 and ZH207/2013) and with the French Ministry of Research and the ethical committee “Paris-Sud et Centre” (License number 2017-12 and 2023-20).

#### Human subjects

We recruited n = 18 human subjects (13 females, 5 males) at the University of Zurich, aged between 18 and 55 years. All experiments procedures were performed in accordance with the Research Ethics Committee of ETH Zurich (2017-N-52) and participants provided written informed consent prior to participation in the study. n = 4 subjects were excluded (3 females and 1 male) because of unstable pitch (bimodal distribution). None of the participants reported being either a professional singer or having absolute pitch. n = 3 participants were recorded before the other participants in a preliminary study using a slightly different experimental design (indicated below). We did not see any systematic difference in those participants and thus decided to include them in the analysis (total n = 14 subjects); when excluding those subjects we obtained qualitatively similar results.

### Method details

#### Song recording and pitch conditioning in birds

We recorded audio with a wall-attached microphone (Audio-Technica Pro4 and 2). The signal was amplified, filtered, and digitized at 32 kHz. We controlled sound acquisition and real-time pitch feedback with a custom LabView (National Instruments, Inc.) program (https://gitlab.switch.ch/hahnloser-songbird/published-code/recoorder).

Pitch conditioning: We trained a two-layer neural network to detect the beginning of a syllable containing a harmonic stack.^77^ In detected syllables, we chose a suitable 16-24 ms segment and evaluated the fundamental frequency (pitch) using the Harmonic Product Spectrum algorithm.^35^^,^^78^ When the pitch was either above or below a manually set threshold, we broadcast a 50-60 ms white-noise (WN) stimulus through a loudspeaker.

#### Modeling framework

##### Latent reinforcement learning equations

We assumed that the pitch *p*_*t*_ of syllable rendition *t* =1,…,*T* is the sum of three sources of variability:

(Equation 1) $$p_{t}=\epsilon_{t}+b_{t}+V_{t}\text{,}$$

where

• $\epsilon_{t}∼P\left(\epsilon_{t}\right)=N\left({0,\sigma }_{\epsilon }^{2}\right)$ are **explorations** attributed to the learner, forming independent and identically distributed (iid) Gaussian random variables with unknown variance $\sigma_{\epsilon }^{2}$,
• *b*_*t*_ forms the learned **pitch bias**, and
• *V*_*t*_ is the sum of **unreinforced behavioral components** capturing the birds’ repertoire (or ‘other behavioral components’).

The learner’s **policy** *π*_*b*_ is to generate an additive motor contribution a=ϵ+b with probability $\pi_{b}=p\left(a|b\right)=P\left(\epsilon \right)$, where we have omitted the time index *t* for simplicity.

The learner performs policy gradient ascent.^3^ That is, it changes the policy *π*_*b*_ along the gradient of a total **benefit function** *J* composed of the expected reward *R*, offset by a cost proportional to the squared bias (to annihilate the bias is a simple model of song maintenance):

$$J\left(b\right)=\int daP\left(a|b\right)\left(R-\frac{\delta }{2\alpha }b^{2}\right)\text{,}$$

where *δ* and *α* are parameters (*α* we will interpret as the learning rate). As for the reward, we set *R*_*t*_ = 0 on escape trials and *R*_*t*_ = −1 on hit trials. We obtained similar results when we changed the definition of the reward to either $R_{t}=\left\{1,0\right\}$ or {1,−1} on {escape,hit}.

The **gradient** ∇_*b*_*J* is given by

$$\nabla_{b}J\left(b\right)=\frac{1}{\sigma_{\epsilon }^{2}}\left[\int P\left(\epsilon \right)\epsilon Rd\epsilon -\frac{\delta }{\alpha }b\right]\text{,}$$

where we have used that ϵ=a−b.

By updating the pitch bias in the direction of the policy gradient, $b\to b+\alpha \sigma_{\epsilon }^{2}\nabla_{b}J$, we obtain the following iterative update for the bias (after replacing the integral over the density *P*(*ϵ*) by samples):

$$b_{t}=b_{t-1}+\alpha R_{t-1}\cdot \epsilon_{t-1}-\delta b_{t-1}\text{.}$$

We add a small iid noise source *η*_*t*_ with unknown variance $\sigma_{\eta }^{2}$ to this equation (to perform parameter estimation using the formalism of a Kalman filter, see below), resulting in:

(Equation 2) $$b_{t}={\left(1-\delta \right)b}_{t-1}+\alpha R_{t-1}\cdot \epsilon_{t-1}+\eta_{t}\text{,}$$

which is the **Pitch Learning Equation**.

We see that according to Equation 2, the learned motor bias *b*_*t*_ performs a leaky correlation between explorations and rewards. The parameter *δ* acts as a time constant that defines how fast the bias decays towards zero (pitch maintenance). Without rewards ($R_{t}\equiv 0)$, the bias acts like a noise source that is driven by *η*_*t*_; since we strictly impose δ<1, this noise source is non-white and so satisfies the presumed efficiency of reinforcement learning—only explorations are white).

We kept modifying the behavioral components of a bird’s repertoire *V*_*t*_ in Equation 1 by trial and error until the **estimated explorations** ${\hat{\epsilon }}_{t}$ were close to ideal (i.e., the model was self-consistent, Figures 2C–2G).

We obtained satisfactory self-consistency when using the behavioral components

(Equation 3) $$V_{t}={p^{*}+c}_{t}+d_{t}+o_{t}\text{,}$$

as detailed in the following.

**Target pitch** *p*^∗^ is the song memory. During free unconstrained singing, syllable pitch has a stable *p*^∗^ i.e. mean pitch is stable from one day to the next.

**Colored noise** *c*_*t*_: We account for slow fluctuations in the pitch dynamics with a **colored noise component** *c*_*t*_ that obeys a random walk: $c_{t}=\left(1-\tau \right)c_{t-1}+\varepsilon_{t}\text{,}$ where *τ* is an unknown time constant and $\varepsilon_{t}∼N\left(0,\sigma_{\varepsilon }^{2}\right)$ is a Gaussian random variable with unknown variance $\sigma_{\varepsilon }^{2}$. The autocorrelation function of colored noise decays exponentially with a decay constant set by the **time constant** *τ* and so for *τ* < 1 is strictly different from the delta-function like autocorrelation of white noise.

**Circadian pattern** *d*_*t*_: Zebra finch song exhibits circadian patterns manifest as repetitive pitch oscillations with a 24-h period. We modeled this pattern *d*_*t*_ as a piecewise linear function constructed as follows.

We first introduced an auxiliary time function *h*(*t*) equal to the time-of-day of trial *t*, expressed as a fraction of time since midnight (0≤h(t)<1). Next, we divided the daytime (during which the lights in the chamber are on) into *N*_*D*_ = 6 time periods $\left[H_{i},H_{i+1}\right]$ ($i=1,\ldots ,N_{D}$) such that on average (across all days) the same number of syllable renditions falls into each period (yielding more bins in the morning because birds tend to sing more in the morning). Thus, the time points $H_{1},\ldots ,H_{N_{D}+1}$ (in fractions of a day) are such that the number of renditions in the interval $H_{j}\leq h\left(t\right)<H_{j+1}$ are equal for all $j=1,\ldots ,N_{D}$ (Figure S1A). The daily pattern *d*_*t*_ is then defined as

$$d_{t}=\sum_{j=1}^{N_{D}}D_{j}\theta_{h\left(t\right)}^{j},\text{where}\;\theta_{h\left(t\right)}^{j}=\left\{ \begin{array}{cc} 1-\frac{h\left(t\right)-H_{j}}{H_{j+1}-H_{j}} & \text{if}\;{\;H}_{j}\leq h\left(t\right)<H_{j+1}\\ \frac{h\left(t\right)-H_{j-1}}{H_{j}-H_{j-1}} & H_{j-1}\leq h\left(t\right)<H_{j}\\ 0 & \text{otherwise} \end{array}\right.$$

and where *D*_*j*_ is a **circadian coefficient** corresponding to the circadian change in pitch at time *H*_*j*_. We tested different number of time periods *N*_*D*_ between 3 and 12 and a linear time binning and found that our results do not qualitatively depend on this parameter.

**History dependence** *o*_*t*_: The pitch of a syllable is influenced by the history of singing, i.e. how much the bird recently sang. We assume that the number of times the bird produced a target syllable (i.e., a placeholder of the number of song motifs) in the last *x* seconds has an additive effect on pitch. We modeled the history dependence of pitch as a fixed pitch offset $o_{t}=O_{{1+n}_{h}\left(t\right)}\text{,}$ where $n_{h}\left(t\right)\in \left\{1,\ldots ,N_{h}\right\}$ is the **number of target syllables within** *x* **seconds preceding rendition** *t*.

We tested different history horizons *x* (1 s, 2 s, 3 s, 5 s, 10 s, and 60 s) and different **history quantization** *N*_*h*_ used as a ceiling: $n_{h}\left(t\right)\to \min\left(n_{h}\left(t\right),N_{h}\right)$. We compared history model variants using a Bayesian information criterion (BIC) and found that the lowest average BIC was achieved for *x* = 2 seconds and *N*_*h*_ = 5 (average BIC = $2.5*10^{5}\pm 1.1*10^{5}$, n=18 birds, Figure S1C), which we used throughout our experiments. For birds that produced at most 3 target syllables in the last 2 seconds, we set *N*_*h*_ to 3 + 1 = 4.

##### Alternative models

###### Classical RL model

For the model encapsulating an optimal policy-gradient strategy without unreinforced behavioral patterns, we set $V_{t}=p^{*}$ and fit the data using the equation $p_{t}=p^{*}+\epsilon_{t}+b_{t}$.

###### Efference copy model

This model is the same as the classical RL model but learning is achieved by replacing $\alpha R_{t-1}{\cdot \epsilon }_{t-1}$ with $\alpha R_{t-1}\cdot \left(\epsilon_{t-1}+V_{t-1}\right)$ in Equation 2.

###### Pitch error model

Learning is achieved by replacing $\alpha R_{t-1}{\cdot \epsilon }_{t-1}$ with $\alpha \left(R_{t-1}-\bar{R_{t-1}}\right)\cdot \left(p_{t-1}-p^{*}\right)$ in Equation 2 where $\bar{R_{t}}=\frac{1}{20}\sum_{i=t-20}^{t-1}R_{i}$ is the running average of rewards over the proceeding 20 renditions.

Everything else is done analogously as for the classical RL model explained above. Both the efference copy model and the pitch error model align with the latent RL framework and can be viewed as extensions of the basic latent RL model. However, despite their added complexity, neither variant significantly improved the model fits (see Figure 3).

##### The model written as a Kalman filter

Mathematically, latent RL models *p*_*t*_ as a linear dynamical system with input *u*_*t*_ that depends on some aspects of syllable renditions *t* and t−1 and on the reward at t−1. To fit the maximum 18 model parameters ($p^{*},\alpha ,\delta ,\tau ,\sigma_{\epsilon }^{2},\sigma_{\varepsilon }^{2},D_{t},O_{k})$ to the behavioral data and estimate the hidden states (${\epsilon_{t},b}_{t},c_{t})$ of this model, we write it as a (linear) Kalman filter with time-dependent coefficients and inputs:

(Equation 4) $$p_{t}=Hx_{t}+g_{t}$$

(Equation 5) $$x_{t}=C_{t}{\tilde{x}}_{t-1}+q_{t}\text{,}$$

where *p*_*t*_ (the pitch of rendition *t*) is the scalar observation, *H* = [1, 1] is the **observation matrix**, *x*_*t*_ is the **latent state**, *C*_*t*_ is the **transition matrix**, ${\tilde{x}}_{t-1}=\left[\begin{array}{c} x_{t-1}\\ u_{t} \end{array}\right]$ is a combination of the latent state and a time-dependent **input vector** *u*_*t*_, and *q*_*t*_ and *g*_*t*_ are **Gaussian iid noises** with (diagonal) **covariance matrices** *Q* and *G*. These noises need to be non-zero to compute the optimal parameters with the expectation maximization algorithm (see Supplement ‘Parameter and state estimation’). In practice, we fixed the output noise covariance *G* to be very small, $g_{t}∼N\left(0,10^{-4}\right)$. It follows that we estimated the explorations *ϵ*_*t*_ not as the output noise of the Kalman filter but as the hidden-state noise part of *q*_*t*_.

Note, we also tested a version where the explorations *ϵ*_*t*_ correspond to the output noise *g*_*t*_ and we obtained qualitatively similar results. However, this latter version requires some approximations (see Supplement). Note also, it is possible to compute the best fit parameters without the Kalman filter formulation (and without the addition of small noise terms) but using nonlinear programming solvers.

##### Baseline model

To model baseline data (no feedback, $R_{t}\equiv 0$), we set the bias *b*_*t*_ to zero. Equations 1, 2, and 3 map to the Kalman filter Equations 4 and 5 with $x_{t}=\left[\begin{array}{c} c_{t}\\ {p^{*}+d}_{t}+o_{t} \end{array}\right]$, $q_{t}=\left[\begin{array}{c} \varepsilon_{t}\\ \epsilon_{t} \end{array}\right]∼N\left(0,Q\right)$ with $Q=\text{diag}\left(\sigma_{\varepsilon }^{2},\sigma_{\epsilon }^{2}\right)$, and a time-dependent input vector $u_{t}={\left[\begin{array}{cc} {\left\{\theta_{h\left(t\right)}^{j}\right\}}_{j=1,\ldots ,N_{D}+1} & {\left\{\delta_{t}^{j}\right\}}_{j=1,\ldots ,N_{h}} \end{array}\right]}^{\prime }$ of dimension $N_{D}+1+N_{h}$. The transition matrix *C*_*t*_ is not time dependent and given by $C=\sum_{i}\theta_{i}C^{i}$ with

$${\left(C^{1}\right)}_{kl}=\left\{ \begin{array}{cc} 1 & \text{if}\;k=1\;\text{and}\;l=1\\ 0 & \text{otherwise} \end{array}\right.\text{,}$$

$${\left(C^{1+j}\right)}_{kl}=\left\{ \begin{array}{cc} 1 & \text{if}\;k=2,l=1+j\\ 0 & \text{otherwise} \end{array}\right.,\text{for}\;j=1,\ldots ,1+N_{D}+N_{h}\text{,}$$

and $\Theta =\left\{\left(1-\tau \right),p^{*}+D_{1},\ldots ,p^{*}+D_{N_{D}+1},O_{1},\ldots ,O_{N_{h}}\right\}$. We set $\theta_{2,\ldots ,N_{D}+2}$ to $p^{*}+D_{1},\ldots ,p^{*}+D_{N_{D}+1}$ to enforce circadian patterns with zero average and fitted the model parameters *θ*_*i*_ and $(\sigma_{\varepsilon }^{2},\sigma_{\epsilon }^{2}$) as described in Supplement ‘Parameter and state estimation’.

##### Reinforcement model

We estimated the learning-related parameters Θ={(1−δ),α} and ($\sigma_{\epsilon }^{2}$, $\sigma_{\eta }^{2}$) by first fitting the baseline data and fixing the estimated baseline parameters during the feedback period except the exploration variance $\sigma_{\epsilon }^{2}$ that we estimated a second time for the purpose of testing the self-consistency of exploration estimation, Figure 2G.

When we include the learner’s bias *b*_*t*_ during the feedback period (now non-zero) and fix the parameters estimated on the baseline data, the pitch values obey the Kalman filter equation with $x_{t}=\left[\begin{array}{c} b_{t}\\ c_{t}\\ p^{*}+d_{t}+o_{t} \end{array}\right]$, $g_{t}∼N\left(0,10^{-4}\right)$, $q_{t}=\left[\begin{array}{c} \eta_{t}\\ \varepsilon_{t}\\ \epsilon_{t} \end{array}\right]$ with corresponding diagonal *Q*, and $u_{t}=\left[\begin{array}{c} c_{t}+d_{t}\\ c_{t-1}+d_{t-1} \end{array}\right]$. The second dimension of *u*_*t*_ is needed to estimate $\epsilon_{t-1}={\left(x_{t-1}\right)}_{3}-\left(c_{t-1}+d_{t-1}\right)$ appearing in the equation for *b*_*t*_, where ${\left(x_{t-1}\right)}_{3}$ stands for the third component of the vector *x*_*t*-1_. Here, the transition matrix *C*_*t*_ is time dependent because of the time dependence of reward ($R_{t}=\left\{-1,0\right\}$). The transition matrix satisfies $C_{t}=C^{0}+\sum_{i}\Theta_{i}C_{t}^{i}$ with ${\Theta \;}_{i}=\left\{\left(1-\delta \right),\alpha \right\}$ and

$${\left(C_{t}^{1}\right)}_{kl}={\left(C^{1}\right)}_{kl}=\left\{ \begin{array}{cc} 1 & \text{if}\;k=1\;\text{and}\;l=1\\ 0 & \text{otherwise} \end{array}\right.\text{,}$$

$${\left(C_{t}^{2}\right)}_{kl}=\left\{ \begin{array}{cc} {-R}_{t-1} & \text{if}\;k=1\;\text{and}\;l=5\\ R_{t-1} & \text{if}\;k=1\;\text{and}\;l=3\\ 0 & \text{otherwise} \end{array}\right.\text{,}$$

$${\left(C^{0}\right)}_{kl}=\left\{ \begin{array}{cc} 1-\tau & \text{if}\;k=2\;\text{and}\;l=2\\ 1 & \text{if}\;k=3\;\text{and}\;l=4\\ 0 & \text{otherwise} \end{array}\right.\text{.}$$

Note that there is no straightforward method to estimate all model parameters at the same time. When the learner’s bias *b*_*t*_ is non-zero, we would either have to add another iid noise term (i.e., one for exploration and the other for learning the parameters *D*_*t*_ and *O*_*k*_) or have to deal with nonlinearities arising from multiplicative parameter combinations (because $\alpha R_{t-1}{\cdot \epsilon }_{t-1}$ depends both on *α* and the parameters *D*_*j*_ and *O*_*k*_). Another possibility is to make simplifying approximations, i.e., to assume that the circadian rhythm does not change much between successive renditions (i.e., setting $d_{t}\approx d_{t-1}$) and that τ is small such that (1−τ)≈1. When we tested fits using these simplifying approximations and estimating all parameters simultaneously, we obtained qualitatively similar results compared to the results shown in the figures. Finally, another alternative would be to estimate parameters by setting ${q_{t}}^{\left(3\right)}∼N\left(0,10^{-4}\right),u_{t}=\left[\begin{array}{c} p^{*}+c_{t}+d_{t}\\ p_{t-1} \end{array}\right]\text{,}$ and $g_{t}∼N\left(0,\sigma_{\epsilon }^{2}\right)\text{,}$ in which case $\epsilon_{t-1}$ would be estimated as $p_{t-1}-r_{t}-c_{t}{-d}_{t}-o_{t}$. We also obtained similar results when trying this alternative, i.e., self-consistent fits with very low AMI.

##### Mutual average information

Unless specified otherwise, we calculated the mutual average information (MAI) on 50 log-spaced rendition lags (integers) from 10^0^ to 10^3^ using the MATLAB (Mathworks Inc) function mai (https://www.mathworks.com/matlabcentral/fileexchange/880-mutual-average-information). To account for the non-zero MAI of a finite time series, we computed the average MAI of three randomly drawn Gaussian iid. time series of the same length as the pitch trajectory in each bird (plotted as dashed lines in Figure 2F). We then normalized the MAI for all birds individually such that zero corresponds to the average MAI over all 50 log-spaced rendition lags of the three random time series and one to the bird’s MAI at lag 1 (Figure 2G).

##### Generative model

We validated the fitted Kalman model in terms of the artificial data it generates. For each bird and corresponding fitted parameters, we let the model generate the same number of pitch renditions as produced by the bird, using the renditions’ timestamps. As we did in the experiments, for each simulated day, we set the reward threshold to the median of the simulated pitch values of the previous day. We simulated each birds’ trajectory 100 times; these we compared with the true trajectory after smoothing both simulations and true trajectories via a running average of the last 50 renditions (see Figures 3E, 3F, S3A, and S3B).

To test whether the different models can explain the amount of learning we observe in birds, we computed the average pitch on the last day of learning. To compare birds, we normalized the pitch between the threshold on the first day of learning (=0) and the threshold on the last day of learning (=1). Birds achieve on average a 0.89 ± 0.21 shift corresponding to 89% of the difference between the two thresholds. We compared the shifts on the last day of different models to the observed shift that birds achieved using a paired two-sided t-test (Figure 3G).

For each smoothed simulation *i* (*i* = 1,…,100), we calculated the **root mean squared error (RMSE)** *E*_*i*_, i.e., the root-mean square (RMS) pitch deviation from the smoothed observed behavior. We then used the average over all simulations as a model quality for each bird, the lower the average RMSE the better (Figure 3H).

To compare the latent learner model with classical RL models where all behavioral variability is exploited during learning and other learning mechanisms, we estimated the model parameters of the alternative models (see above). We compared the latent with the classical RL model and the different reinforcement mechanisms in terms of average RMSEs via a paired two-sided t-test (we tested whether their average RMSE are equal). Furthermore, we also compared their MAI at lag 1 using a paired two-sided t-test.

We expect that birds continuously learn to improve their behavior and a good policy gradient strategy should generate smooth learning i.e. a smooth bias *b*_*t*_. To estimate smoothness of the estimated bias *b*_*t*_ independent of how much the bird sung, we first resampled the *b*_*t*_ of each day to 1000 timestamps using the MATLAB function resample (example in Figure 3I). We then quantified smoothness as the mean squared second derivative (MS2D) of the resampled pitch bias (the smoother the lower the MS2D) and compared the MS2D of the latent RL model to the other models using a paired two-tailed t-test, Figure 3J.

##### Exploration variance

For each bird, we computed the variance of each estimated behavioral component (${\hat{\epsilon }}_{t}$, ${\hat{c}}_{t},{\hat{d}}_{t}$, ${\hat{o}}_{t}$) during baseline and illustrate their contribution to the sum in Figure 4A.

To test how much data is needed to estimate the exploration variance and whether it is possible to do so only during baseline (no feedback), we compute the best fit model parameters (including $\sigma_{\epsilon }^{*}$, ∗ indicating best fit) using either 1, 3, or 5 days of baseline data (7 birds were excluded for 5-day estimation because of data unavailability). We then compared the best fit exploration standard deviation normalized to the target pitch *p*^∗^ during baseline $\sigma_{\text{BL},\epsilon }^{*}$ to the estimated variance $\sigma_{\epsilon }^{*}$ during and after the feedback period (excluding baseline).

Across birds, the Pearson correlations *ρ* between $\sigma_{BL,\epsilon }^{*}$ and $\sigma_{\epsilon }^{*}$ were large regardless of the number of baseline days included (Figure 4B):

• 1 day: *ρ* = 0.90, *p* = 4.9 ∗ 10^-7^ (n = 18birds).
• 3 days: *ρ* = 0.91, *p* = 1.1 ∗ 10^-7^ (n = 18 birds).
• 5-days: *ρ* = 0.91, *p* = 1.2 ∗ 10^-4^ (n = 11 birds).

We fitted the BL vs feedback exploration variances in Figures 4B and 6I using Matlab’s regress function, determining the *p* value at which the unit slope *m* = 1.0 fell outside the estimated confidence interval, which amounts to a Student’s t-test.

##### Contribution to learning

We computed the Pearson correlation coefficient between the best-fit learning rate and the 24-h pitch improvement and the exploration standard deviation $\sigma_{\epsilon }^{*}$ and the 24-pitch improvement. Birds usually learn faster in the early days of learning and then slow down after a few days. We therefore quantified 24-h pitch improvements as the difference between the mean pitch of the first 100 morning syllables of consecutive days averaged over the first 3 days of learning.

To compute the contribution of exploration $\sigma_{\epsilon }^{*}$, the learning rate *α*^∗^, and the motor fluctuations ${\hat{\sigma }}_{V}=\text{std}\left({\hat{V}}_{t}\right)$, we fitted a linear mixed effect model to average daily pitch improvement Δ*P*:

${\Delta P\;}^{b}=e*{\sigma_{\epsilon }^{*}}^{b}+a*{\alpha^{*}}^{b}+c*{{\hat{\sigma }}_{V}}^{b}+\varepsilon^{b}$ where *b* indicates the different birds, *ε*^*b*^ is a random effect for each bird and *e*, *a*, and *c* are the fixed effects for exploration, learning rate, and motor fluctuations. To display the contributions of each fixed term to learning, we multiplied the fitted fixed effect terms with the average predictor over all birds, i.e. $e*\text{mean}\left({\sigma_{\epsilon }^{*}}^{b}\right)$ etc. and divided it by the average learned shift Δ*P*^*b*^ and multiplied by 100, Figure 4I. The p-values of the fixed terms are reported in the paper and indicated as stars in the figure.

#### LMAN lesions

We performed surgeries under general anesthesia (0.7-1.2% isoflurane, induction at 2%); providing analgesia with lidocaine delivered both globally (subcutaneous injection of 0.05 ml solution) and locally along the planned skin incision (0.05 g Emla® creme). We performed craniotomies above LMAN (1.7 mm lateral to the midline and 5.3 mm anterior to the confluence of sinuses, measured at a 35-degree angle of the flat anterior part of the skull). In each hemisphere, we assessed the center and extent of LMAN electrophysiologically using a single Tungsten wire electrode of 0.8-1.2 MΩ impedance (Micro Probe, Inc.).

We lesioned LMAN by pressure injecting (Picosprizter® III, Parker) ibotenic acid at 3-5 sites in LMAN (the center and 250 μm medially and laterally and/or anterior-posterior depending on electrophysiological response) via borosilicate glass pipettes (BF-120-69-10, Sutter instrument), as in.^35^

##### Histology

We euthanized 25 birds and perfused them with phosphate-buffered saline (PBS), followed by 4 % paraformaldehyde. We removed the cerebrum and kept the brain in paraformaldehyde for at least 24 hours before further processing. We embedded each hemisphere individually in agar before cutting it into 80-μm sagittal sections. We Nissl stained all slices with 0.3 % cresyl violet acetate solution.

##### Quantification of lesion volume

We identified LMAN in sagittal brain slices by its large cell nuclei and its location in the nidopallium dorsal to Area X and between the lamina frontalis superior and the lamina pallio-subpallialis,^79^^,^^80^ see e.g. Figures S5A and S5B.

We estimated the lesioned LMAN volume as a fraction of the average intact LMAN volume in unmanipulated (control) adult males. To estimate the uni-hemispheric LMAN volume $V_{con\;}$ in control birds (n=8), in each sagittal slice we quantified the area of LMAN and multiplied that with the section thickness. The summed volumes we averaged across (n=16) hemispheres, yielding a reference LMAN volume of $V_{cont\;}=0.12\pm 0.02\;{\mu m}^{3}$, which is within the published range of $0.099{\;\mu m}^{3}$^81^ and $0.2\;{\mu m}^{3}$.^82^ We quantified the unlesioned LMAN volume $V_{remaining}$ in each (n=17) manipulated bird and hemisphere in the same way (see Figures S5C and S5D).

We then computed the fraction LMAN volume that was lesioned in the right hemisphere *F*_*les*,*r*_ as the difference between the remaining LMAN volume and the reference volume, normalized by the reference volume:

$$F_{les,r\;}=\frac{V_{con\;}-V_{rem,r}}{V_{con\;}}\;\text{.}$$

We similarly computed the fraction *F*_*les*,*l*_ of lesioned LMAN volume in the left hemisphere. In one hemisphere, the numerator was negative and so we set the fraction to zero.

The final fraction *F*_*les*_ of lesioned LMAN volume in each bird we estimated as the average across the right and left hemispheres:

$$F_{les\;}=\frac{F_{les,r}+F_{les,l}}{2}\;\text{.}$$

The average fraction of lesioned LMAN volume was $\left\langle F_{les}\right\rangle =61\pm 28\%$ (SD, n=17 birds, range 0−100%, Figure S5D). We missed LMAN completely in both hemispheres in n = 3 birds. We included these 3 birds in the analysis except when indicated (Figure 5G) because we used LMAN lesion volume as a predictor for the lesion effect (Figure 5F). Excluding the n = 3 birds with 0% LMAN lesioned did not qualitatively change the results.

#### Pitch conditioning in humans

Audio signals were acquired with a microphone (SM6 model from Røde) placed in front of the subjects. The signals were amplified, filtered and digitized at 32 kHz with the same system we used for birds. Subjects were instructed to repeat the syllable “Da” at a speed of about 2 “Da's” per second. For all except the first 3 participants, a metronome was shown on the screen to help them maintain a steady pace. We detected the onset of a syllable by thresholding sound amplitude (RMS sound waveform), the threshold was kept constant at a level well above the noise level of the room.

During a test session, subjects were instructed to adjust the sound amplitude of their voice (displayed on the screen) such that it fell below the threshold between vocalizations and by a factor of 4-6 above the threshold during the vocalizations. We evaluated pitch in a 16-ms window at a fixed latency of 88 ms to vocalization onsets.

The experiment was conducted in short sessions interleaved by breaks. During the breaks, participants were encouraged to drink water and/or take a lozenge for the throat.

Participants performed first 1-3 baseline sessions of roughly 6 minutes each and then 1-2 conditioning sessions (the first 3 participants performed baseline and conditioning sessions on different days). Before each conditioning session, participants were asked to roll a dice to determine the initial white-noise contingency which was unknown to them (1-3: WN on low pitch; 4-6: WN on high-pitch). On the second conditioning session we inverted the contingency independent of their second roll.

During a conditioning session, we first recorded 1 minute of data without conditioning, then 3 minutes with white noise conditioning, and again 2 minutes without conditioning (in one participant we omitted a white-noise free period after conditioning). We set the white-noise threshold to the median pitch of the 20 most recent renditions. We automatically adjusted the threshold when the escape rate during the last 20 renditions was above 80%. We also adapted the threshold when the escape rate was below 5%, to prevent subjects from getting stuck at pitch values where WN becomes uninformative (where all renditions trigger WN).

Participants were given the following initial instructions (session numbers dependent on participant): “During the third and fourth sessions, you will sometimes hear a white noise sound after your Da. When you hear the white noise sound, please try to stay as calm as possible and continue saying Da as before. The goal is to avoid this white noise sound but stay as relaxed as possible.” The experimenter verbally repeated the instructions before the second conditioning session.

To quantify pitch changes in either the baseline, feedback, or recovery period (Figure 6C), we compared the average pitch of the last 50 renditions to the average pitch of the first 50 renditions in that period. The feedback period corresponds to the trials in the conditioning session on which the threshold was different from zero and the recovery period corresponds to all renditions on which the threshold was set back to zero (participants could not trigger white noise anymore).

We also compared pitch changes during baseline and feedback periods at identical rendition lags since beginning of the period (i.e. if the first 50 renditions during the feedback period were renditions 201-250, then the same rendition lags were used to average baseline pitch). We tested for differences in average pitch across periods using paired one-tailed t-tests over subjects and we tested for non-zero pitch changes during the feedback period using two-tailed t-tests. We tested for correlations between exploration variances associated with baseline and WN sessions in terms of the Pearson correlation coefficient.

Humans shifted their pitch away from the WN zone as expected: the pitch difference between the first and last 50 utterance renditions of a session was *d*′ = 4.8 ± 7.8 (*p* = 0.02, one-tailed t-test, *tstat* = 2.30, *df* = 13), and similarly the pitch of the last 50 renditions differed from the pitch of the corresponding 50 renditions of the preceding baseline session by *d*′ = 5.1 ± 7.9 (*p* = 0.03, two-tailed paired t-test, *tstat* = 2.43, *df* = 13, Figure 6C). After cessation of WN delivery, during the short recovery time period we provided, subjects tended to revert their pitch towards baseline by *d*′= −1.5 ± 3.3 (*p* = 0.07, one-sided t-test, *at* = −1.62, *df* = 12, excluding one subject who was not given a recovery time period). The observed changes in pitch were not only driven by the three subjects with the strongest escape response, as a significant effect was retained after removing those three subjects.

To estimate explorations, we simulated a model without circadian pattern and history dependence because of the brevity of the baseline and WN sessions. Because of considerable pitch variability among sessions, we analyzed explorations merely in the last baseline session. For all subjects except two, we analyzed the first conditioning session because during the second conditioning session subjects tended to get distracted or start to play around with the system by strongly modulating their voice. Two subjects did not understand the task correctly in the first conditioning session, which is why we analyzed data from their second session.

##### Pitch steps

We detected discontinuities or steps in human pitch dynamics (Figure S6) when the average of the *n*_*s*_ = 20 pitch values before a given rendition differed from the average of the 20 pitch values following (and including) that rendition by more than a factor Θ of the average pitch across all 41 renditions: A step at rendition *i* was detected when the pitch step satisfied $\left|p_{i}^{l}-p_{i}^{r}\right|>\;\Theta p_{i}^{m}$, where $p_{i}^{l}={\left\langle p_{i}\right\rangle }_{\left[i-20,i\right]}\text{,}$ $p_{i}^{r}={\left\langle p_{i}\right\rangle }_{\left[i,i+20\right]},p_{i}^{m}={\left\langle p_{i}\right\rangle }_{\left[i-20,i+20\right]}\text{.}$ We detected discontinuities using Θ = 0.05, 0.01, or 0.1.

Once we detected a discontinuity, we removed it from the pitch trajectory to look for more discontinuities until none was left in the given session. We iteratively eliminated the largest pitch discontinuity in terms of the maximum pitch step. Elimination was done by adding the step size to all subsequent renditions: if the largest discontinuity was at rendition *i*, then we applied the transformation $p_{j>i}=p_{j>i}+\left|p_{i}^{l}-p_{i}^{r}\right|$ to all following pitches, see Figure S6.

We then analyzed the rate of pitch discontinuities, in particular the rate *rA* following WN experience: A pitch discontinuity followed WN experience if WN was delivered on any of the 20 renditions preceding the discontinuity. We calculated this discontinuity rate as $rA=\frac{nD_{WN}}{T}$, where *nD*_*WN*_ is the number of discontinuities following WN experience and *T* the number of pitch renditions in the session (we deliberately ignored discontinuities at session boundaries).

We compared *rA* to the chance-level rate *rC* that assumes no relationship between WN and discontinuities. This latter rate we calculated as $rC=\frac{nWN}{T}\text{,}$ where *nWN* is the number of renditions preceded by WN. We compared the rates *rA* and *rC* using a test for equality of proportions (i.e., the rates are probabilities per rendition) using the *Z* test statistics defined as $Z=\frac{r_{A}-r_{C}}{\sqrt{r\left(1-r\right)\left(\frac{1}{T}+\frac{1}{n_{D}}\right)}}$, with $r=\frac{nD_{WN}+n_{WN}\;}{nD+T}$ and with *nD* the number of discontinuities in that subject. A subject responded significantly to WN when the difference between their baseline and WN-associated discontinuity rates satisfied |*Z*| > 1.96, corresponding to *p* < 0.05. Subjects that responded to WN by increasing the pitch step rate were said to explore stepwise.

We report results for *n*_*s*_ = 20 and Θ = 5%; the results were quite robust to changes of these parameters, detailed in the following.

• For Θ = 10% (*n*_*s*_ = 20) we observed fewer pitch steps: 3 subjects showed a stepping rate that differed between WN and baseline (plus one subject showed a mere trend: *Z* = 1.67), all four subjects increased their stepping rate during WN, all in agreement with directed stepwise exploration.
• For Θ = 2.5% (*n*_*s*_ = 20) four subjects showed a stepping rate that differs between WN and baseline (plus one subject showed a trend: *Z* = 1.45): Four subjects increased the stepping rate during WN, and one decreased the stepping rate (*Z* = −2.9). Note that at such a small Θ, it can be argued whether the detected steps are true discontinuities, since there are large numbers of them, without showing a clear change in pitch level.
• For *n*_*s*_ = 10 (Θ = 5%), 5/14 subjects showed a significantly different stepping rate during WN, all of them increased the stepping rate, in agreement with directed stepwise exploration.
• For *n*_*s*_ = 10 (Θ = 5%), 6/14 subjects significantly changed the pitch step rate in response to WN (|*Z*| > 1.96), five produced more pitch steps (all in the direction of WN escape), one decreased the pitch step rate (Z = −2.1). The number of steps was in general too small to assess the significance of whether they were aligned with the direction of WN escape or not, we did not observe any clear trend either.

None of the birds produced pitch steps at the level of Θ = 5%. At the finer level of Θ = 2%, we detected some pitch steps. Namely, three of 18 birds significantly changed the pitch stepping rate after experiencing WN (|*Z*| > 1.96), two of them with a higher stepping rate during WN, and one with a lower stepping rate, providing little evidence overall of WN-directed stepwise exploration in birds. Nevertheless, in three birds, the WN-associated steps were biased towards WN escape, suggesting that birds are capable of stepwise exploitation, in line with previous reports.^48^^,^^83^^,^^84^ Thus, human pitch learning might make use of a similar learning mechanism driven by ideal explorations.

### Quantification and statistical analysis

All statistical analyses were performed in MATLAB (MathWorks, R2019b-R2024b). Statistical tests and corresponding exact n values, effect sizes, and p values are provided in the figure legends or results. Unless stated otherwise, n denotes biological replicates, i.e., the number of birds or human subjects, as indicated. Four human subjects were excluded due to unstable (bimodal) pitch distributions, and no birds were excluded except in the 5-day baseline analysis, where insufficient data were available for seven birds. Randomization was not applicable as each bird or subject served as its own control (baseline vs. feedback or pre-lesion vs. post-lesion). Data are expressed as mean ± standard deviation (SD) unless stated otherwise and violin plots show the mean and individual data points.

All the statistical tests are described in detail in the STAR Methods and their result stated in the results section: Parametric tests (two-tailed paired or unpaired t-tests) were used when data was assumed to be normally distributed. Correlations were computed using Pearson’s correlation coefficients. For categorical data (e.g., pitch step rates), equality of proportions was tested using a z-test for proportions. Linear mixed-effects models were fitted using the fitlmematrix function in MATLAB, with bird identity included as a random effect to account for inter-individual variability. Fixed-term p-values are reported in the figure and results. For the lesion study, relationships between lesion extent and behavioral effects were assessed by type-1 linear regression, and confidence intervals were estimated from 100 bootstrap replicates. Significance was defined as *p* < 0.05 (*p* < 0.01, *p* < 0.001 for ∗∗, ∗∗∗, respectively).

### Additional resources

No additional resources were generated or analyzed in this study.

## References

[1] Bhui R., Lai L., Gershman S.J. (2021). Resource-rational decision making. Curr. Opin. Behav. Sci..

[2] Sutton R.S., Barto A.G. (2018).

[3] Williams R.J. (1992). Simple statistical gradient-following algorithms for connectionist reinforcement learning. Mach. Learn..

[4] Silver D., Hubert T., Schrittwieser J., Antonoglou I., Lai M., Guez A., Lanctot M., Sifre L., Kumaran D., Graepel T. (2018). A general reinforcement learning algorithm that masters chess, shogi, and Go through self-play. Science.

[5] Tesauro G. (1994). TD-Gammon, a Self-Teaching Backgammon Program, Achieves Master-Level Play. Neural Comput..

[6] Gadagkar V., Puzerey P.A., Chen R., Baird-Daniel E., Farhang A.R., Goldberg J.H. (2016). Dopamine neurons encode performance error in singing birds. Science.

[7] Hollerman J.R., Schultz W. (1998). Dopamine neurons report an error in the temporal prediction of reward during learning. Nat. Neurosci..

[8] Kim H.R., Malik A.N., Mikhael J.G., Bech P., Tsutsui-Kimura I., Sun F., Zhang Y., Li Y., Watabe-Uchida M., Gershman S.J., Uchida N. (2020). A Unified Framework for Dopamine Signals across Timescales. Cell.

[9] Jeong H., Taylor A., Floeder J.R., Lohmann M., Mihalas S., Wu B., Zhou M., Burke D.A., Namboodiri V.M.K. (2022). Mesolimbic dopamine release conveys causal associations. Science.

[10] Sutton R.S., Barto A.G. (1981). Toward a modern theory of adaptive networks: Expectation and prediction. Psychol. Rev..

[11] Akaishi R., Umeda K., Nagase A., Sakai K. (2014). Autonomous Mechanism of Internal Choice Estimate Underlies Decision Inertia. Neuron.

[12] Akrami A., Kopec C.D., Diamond M.E., Brody C.D. (2018). Posterior parietal cortex represents sensory history and mediates its effects on behaviour. Nature.

[13] Samuelson W., Zeckhauser R. (1988). Status quo bias in decision making | SpringerLink. J. Risk Uncertain..

[14] Watkins C.J.C.H., Dayan P. (1992). Q-learning. Mach. Learn..

[15] Drugowitsch J., Wyart V., Devauchelle A.-D., Koechlin E. (2016). Computational Precision of Mental Inference as Critical Source of Human Choice Suboptimality. Neuron.

[16] Niv Y. (2009). Reinforcement learning in the brain. J. Math. Psychol..

[17] Findling C., Skvortsova V., Dromnelle R., Palminteri S., Wyart V. (2019). Computational noise in reward-guided learning drives behavioral variability in volatile environments. Nat. Neurosci..

[18] Dhawale A.K., Smith M.A., Ölveczky B.P. (2017). The role of variability in motor learning. Annu. Rev. Neurosci..

[19] Therrien A.S., Wolpert D.M., Bastian A.J. (2016). Effective reinforcement learning following cerebellar damage requires a balance between exploration and motor noise. Brain.

[20] van Beers R.J., Haggard P., Wolpert D.M. (2004). The Role of Execution Noise in Movement Variability. J. Neurophysiol..

[21] Chen X., Mohr K., Galea J.M. (2017). Predicting explorative motor learning using decision-making and motor noise. PLoS Comput. Biol..

[22] Therrien A.S., Wolpert D.M., Bastian A.J. (2018). Increasing motor noise impairs reinforcement learning in healthy individuals. eNeuro.

[23] Kao M.H., Doupe A.J., Brainard M.S. (2005). Contributions of an avian basal ganglia-forebrain circuit to real-time modulation of song. Nature.

[24] Wirthlin M., Chang E.F., Knörnschild M., Krubitzer L.A., Mello C.V., Miller C.T., Pfenning A.R., Vernes S.C., Tchernichovski O., Yartsev M.M. (2019). A modular approach to vocal learning: disentangling the diversity of a complex behavioral trait. Neuron.

[25] Andalman A.S., Fee M.S. (2009). A basal ganglia-forebrain circuit in the songbird biases motor output to avoid vocal errors. Proc. Natl. Acad. Sci. USA.

[26] Charlesworth J.D., Warren T.L., Brainard M.S. (2012). Covert skill learning in a cortical-basal ganglia circuit. Nature.

[27] Kawai R., Markman T., Poddar R., Ko R., Fantana A.L., Dhawale A.K., Kampff A.R., Ölveczky B.P. (2015). Motor Cortex Is Required for Learning but Not for Executing a Motor Skill. Neuron.

[28] Kojima S., Kao M.H., Doupe A.J., Brainard M.S. (2018). The avian basal ganglia are a source of rapid behavioral variation that enables vocal motor exploration. J. Neurosci..

[29] Olveczky B.P., Andalman A.S., Fee M.S. (2005). Vocal experimentation in the juvenile songbird requires a basal ganglia circuit. PLoS Biol..

[30] Młynarski W., Hledík M., Sokolowski T.R., Tkačik G. (2021). Statistical analysis and optimality of neural systems. Neuron.

[31] Tumer E.C., Brainard M.S. (2007). Performance variability enables adaptive plasticity of “crystallized” adult birdsong. Nature.

[32] Ali F., Otchy T.M., Pehlevan C., Fantana A.L., Burak Y., Ölveczky B.P. (2013). The Basal Ganglia is necessary for learning spectral, but not temporal, features of birdsong. Neuron.

[33] Charlesworth J.D., Tumer E.C., Warren T.L., Brainard M.S. (2011). Learning the microstructure of successful behavior. Nat. Neurosci..

[34] Hoffmann L.A., Saravanan V., Wood A.N., He L., Sober S.J. (2016). Dopaminergic Contributions to Vocal Learning. J. Neurosci..

[35] Zai A.T., Cavé-Lopez S., Rolland M., Giret N., Hahnloser R.H.R. (2020). Sensory substitution reveals a manipulation bias. Nat. Commun..

[36] Aronov D., Fee M.S. (2012). Natural changes in brain temperature underlie variations in song tempo during a mating behavior. PLoS One.

[37] Wang G., Harpole C.E., Trivedi A.K., Cassone V.M. (2012). Circadian Regulation of Bird Song, Call, and Locomotor Behavior by Pineal Melatonin in the Zebra Finch. J. Biol. Rhythms.

[38] Wood W.E., Osseward P.J., Roseberry T.K., Perkel D.J. (2013). A daily oscillation in the fundamental frequency and amplitude of harmonic syllables of zebra finch song. PLoS One.

[39] Kao M.H., Brainard M.S. (2006). Lesions of an avian basal ganglia circuit prevent context-dependent changes to song variability. J. Neurophysiol..

[40] Singh Alvarado J., Goffinet J., Michael V., Liberti W., Hatfield J., Gardner T., Pearson J., Mooney R. (2021). Neural dynamics underlying birdsong practice and performance. Nature.

[41] Giret N., Kornfeld J., Ganguli S., Hahnloser R.H.R. (2014). Evidence for a causal inverse model in an avian cortico-basal ganglia circuit. Proc. Natl. Acad. Sci. USA.

[42] Hisey E., Kearney M.G., Mooney R. (2018). A common neural circuit mechanism for internally guided and externally reinforced forms of motor learning. Nat. Neurosci..

[43] Xiao L., Chattree G., Oscos F.G., Cao M., Wanat M.J., Roberts T.F. (2018). A Basal Ganglia Circuit Sufficient to Guide Birdsong Learning. Neuron.

[44] Warren T.L., Tumer E.C., Charlesworth J.D., Brainard M.S. (2011). Mechanisms and time course of vocal learning and consolidation in the adult songbird. J. Neurophysiol..

[45] Hampton C.M., Sakata J.T., Brainard M.S. (2009). An avian basal ganglia-forebrain circuit contributes differentially to syllable versus sequence variability of adult Bengalese finch song. J. Neurophysiol..

[46] Thompson J.A., Basista M.J., Wu W., Bertram R., Johnson F. (2011). Dual pre-motor contribution to songbird syllable variation. J. Neurosci..

[47] Toutounji H., Zai A.T., Tchernichovski O., Hahnloser R.H.R., Lipkind D. (2024). Learning the sound inventory of a complex vocal skill via an intrinsic reward. Sci. Adv..

[48] Zai A.T., Stepien A.E., Giret N., Hahnloser R.H.R. (2024). Goal-directed vocal planning in a songbird. eLife.

[49] Zhou B., Hofmann D., Pinkoviezky I., Sober S.J., Nemenman I. (2018). Chance, long tails, and inference in a non-Gaussian, Bayesian theory of vocal learning in songbirds. Proc. Natl. Acad. Sci. USA.

[50] Stepanek L., Doupe A.J. (2010). Activity in a cortical-basal ganglia circuit for song is required for social context-dependent vocal variability. J. Neurophysiol..

[51] Tachibana R.O., Lee D., Kai K., Kojima S. (2022). Performance-Dependent Consolidation of Learned Vocal Changes in Adult Songbirds. J. Neurosci..

[52] Kollmorgen S., Hahnloser R.H.R., Mante V. (2020). Nearest neighbours reveal fast and slow components of motor learning. Nature.

[53] Brudner S., Pearson J., Mooney R. (2023). Generative models of birdsong learning link circadian fluctuations in song variability to changes in performance. PLoS Comput. Biol..

[54] Sankar R., Leblois A., Rougier N.P. (2022). 2022 IEEE International Conference on Development and Learning (ICDL).

[55] Albert S.T., Jang J., Sheahan H.R., Teunissen L., Vandevoorde K., Herzfeld D.J., Shadmehr R. (2021). An implicit memory of errors limits human sensorimotor adaptation. Nat. Hum. Behav..

[56] Churchland M.M., Afshar A., Shenoy K.V. (2006). A central source of movement variability. Neuron.

[57] Dhawale A.K., Miyamoto Y.R., Smith M.A., Ölveczky B.P. (2019). Adaptive regulation of motor variability. Curr. Biol..

[58] van Beers R.J. (2007). The Sources of Variability in Saccadic Eye Movements. J. Neurosci..

[59] Izawa J., Shadmehr R. (2011). Learning from sensory and reward prediction errors during motor adaptation. PLoS Comput. Biol..

[60] Shmuelof L., Krakauer J.W., Mazzoni P. (2012). How is a motor skill learned? Change and invariance at the levels of task success and trajectory control. J. Neurophysiol..

[61] Herzfeld D.J., Vaswani P.A., Marko M.K., Shadmehr R. (2014). A memory of errors in sensorimotor learning. Science.

[62] Sober S.J., Brainard M.S. (2009). Adult birdsong is actively maintained by error correction. Nat. Neurosci..

[63] Wilson R.C., Bonawitz E., Costa V.D., Ebitz R.B. (2021). Balancing exploration and exploitation with information and randomization. Curr. Opin. Behav. Sci..

[64] Ravbar P., Lipkind D., Parra L.C., Tchernichovski O. (2012). Vocal exploration is locally regulated during song learning. J. Neurosci..

[65] Kao M.H., Wright B.D., Doupe A.J. (2008). Neurons in a forebrain nucleus required for vocal plasticity rapidly switch between precise firing and variable bursting depending on social context. J. Neurosci..

[66] Darshan R., Wood W.E., Peters S., Leblois A., Hansel D. (2017). A canonical neural mechanism for behavioral variability. Nat. Commun..

[67] Destexhe A., Rudolph M., Paré D. (2003). The high-conductance state of neocortical neurons in vivo. Nat. Rev. Neurosci..

[68] Van Vreeswijk C., Sompolinsky H. (1996). Chaos in Neuronal Networks with Balanced Excitatory and Inhibitory Activity. Science.

[69] Isomura T., Toyoizumi T. (2016). A local learning rule for independent component analysis. Sci. Rep..

[70] Toyoizumi T., Pfister J.-P., Aihara K., Gerstner W. (2004). Spike-timing dependent plasticity and mutual information maximization for a spiking neuron model. Adv. Neural Inf. Process. Syst..

[71] Salisbury J.M., Palmer S.E. (2016). Optimal Prediction in the Retina and Natural Motion Statistics. J. Stat. Phys..

[72] Marko M.K., Haith A.M., Harran M.D., Shadmehr R. (2012). Sensitivity to prediction error in reach adaptation. J. Neurophysiol..

[73] Robinson F.R., Noto C.T., Bevans S.E. (2003). Effect of Visual Error Size on Saccade Adaptation in Monkey. J. Neurophysiol..

[74] Soetedjo R., Fuchs A.F., Kojima Y. (2009). Subthreshold Activation of the Superior Colliculus Drives Saccade Motor Learning. J. Neurosci..

[75] Wei K., Körding K. (2009). Relevance of error: what drives motor adaptation?. J. Neurophysiol..

[76] Viswanathan G.M., Raposo E.P., da Luz M.G.E. (2008). Lévy flights and superdiffusion in the context of biological encounters and random searches. Phys. Life Rev..

[77] Yamahachi H., Zai A.T., Tachibana R.O., Stepien A.E., Rodrigues D.I., Cavé-Lopez S., Lorenz C., Arneodo E.M., Giret N., Hahnloser R.H.R. (2020). Undirected singing rate as a non-invasive tool for welfare monitoring in isolated male zebra finches. PLoS One.

[78] Noll A.M. (1970). Pitch Determination of Human Speech by the Harmonic Product Spectrum, the Harmonic Sum Spectrum and a Maximum Likelihood Estimate. Proceedings of the Symposium on Computer Processing in Communications.

[79] Lovell P.V., Wirthlin M., Kaser T., Buckner A.A., Carleton J.B., Snider B.R., McHugh A.K., Tolpygo A., Mitra P.P., Mello C.V. (2020). ZEBrA: Zebra finch Expression Brain Atlas—A resource for comparative molecular neuroanatomy and brain evolution studies. J. Comp. Neurol..

[80] Nixdorf-Bergweiler B.E., Bischof H.-J. (2007). A stereotaxic atlas of the brain of the zebra finch, Taeniopygia guttata: with special emphasis on telencephalic visual and song system nuclei in transverse and sagittal sections.

[81] Scharff C., Nottebohm F. (1991). A comparative study of the behavioral deficits following lesions of various parts of the zebra finch song system: implications for vocal learning. J. Neurosci..

[82] Nixdorf-Bergweiler B.E., Lips M.B., Heinemann U. (1995). Electrophysiological and morphological evidence for a new projection of LMAN-neurones towards area X. Neuroreport.

[83] Costalunga G., Carpena C.S., Seltmann S., Benichov J.I., Vallentin D. (2023). Wild nightingales flexibly match whistle pitch in real time. Curr. Biol..

[84] Veit L., Tian L.Y., Monroy Hernandez C.J., Brainard M.S. (2021). Songbirds can learn flexible contextual control over syllable sequencing. eLife.

