# Supplementary material for: Estimating the motor exploration in reinforcement learning
Source: iScience. 2025 Dec 9;29(2):114398. doi: 10.1016/j.isci.2025.114398 (PMC12829137; doi:10.1016/j.isci.2025.114398)
Supplement: Document S1. Figures S1–S6 and supplemental methods [file mmc1.pdf]

iScience, Volume *29*

Supplemental information

Estimating the motor exploration in reinforcement learning

Anja T. Zai, Corinna Lorenz, Shakana Srikantharajah, Nicolas Giret, and Richard H.R. Hahnloser

Supplementary Figures

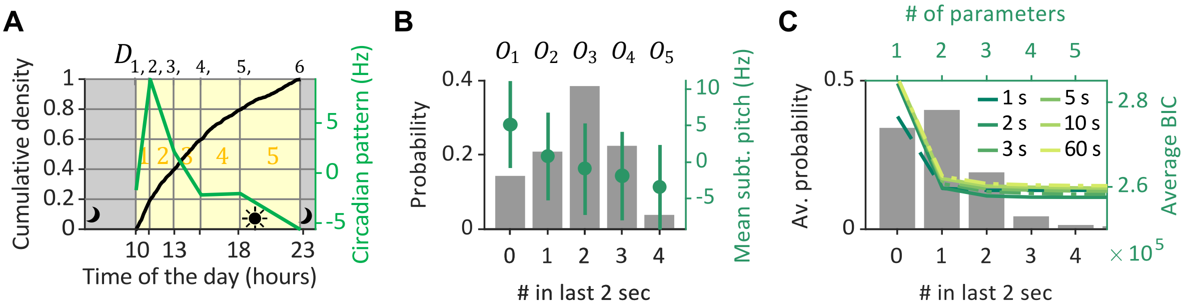

Figure S1: Circadian pattern and history dependence, related to Figure 2. (A) We modeled the circadian pattern (green) as a piecewise linear function here defined by six coefficients $D_{1,\ldots 6}$ spread across 5 daytime bins (yellow shading). Each bin contains 20% of syllable renditions (illustrated by the black normalized cumulative distribution for an example bird during the baseline period; same bird as in Figure 2). (B) The history dependence, we modeled as mean-subtracted pitch offsets O_k (±std, green), where $k = 1, \ldots, 5$ is the number of syllable renditions within the last $\Delta = 2$ seconds (gray bars). Same example bird as in Figure 2. (C) The average Bayesian information criterion (BIC, green lines, $N = 18$ birds) was lowest for $\Delta = 2$ seconds (green solid line). The gray bars show the distribution of syllables that have 0, 1, 2, 3, or 4 target syllables within the last 2 seconds.

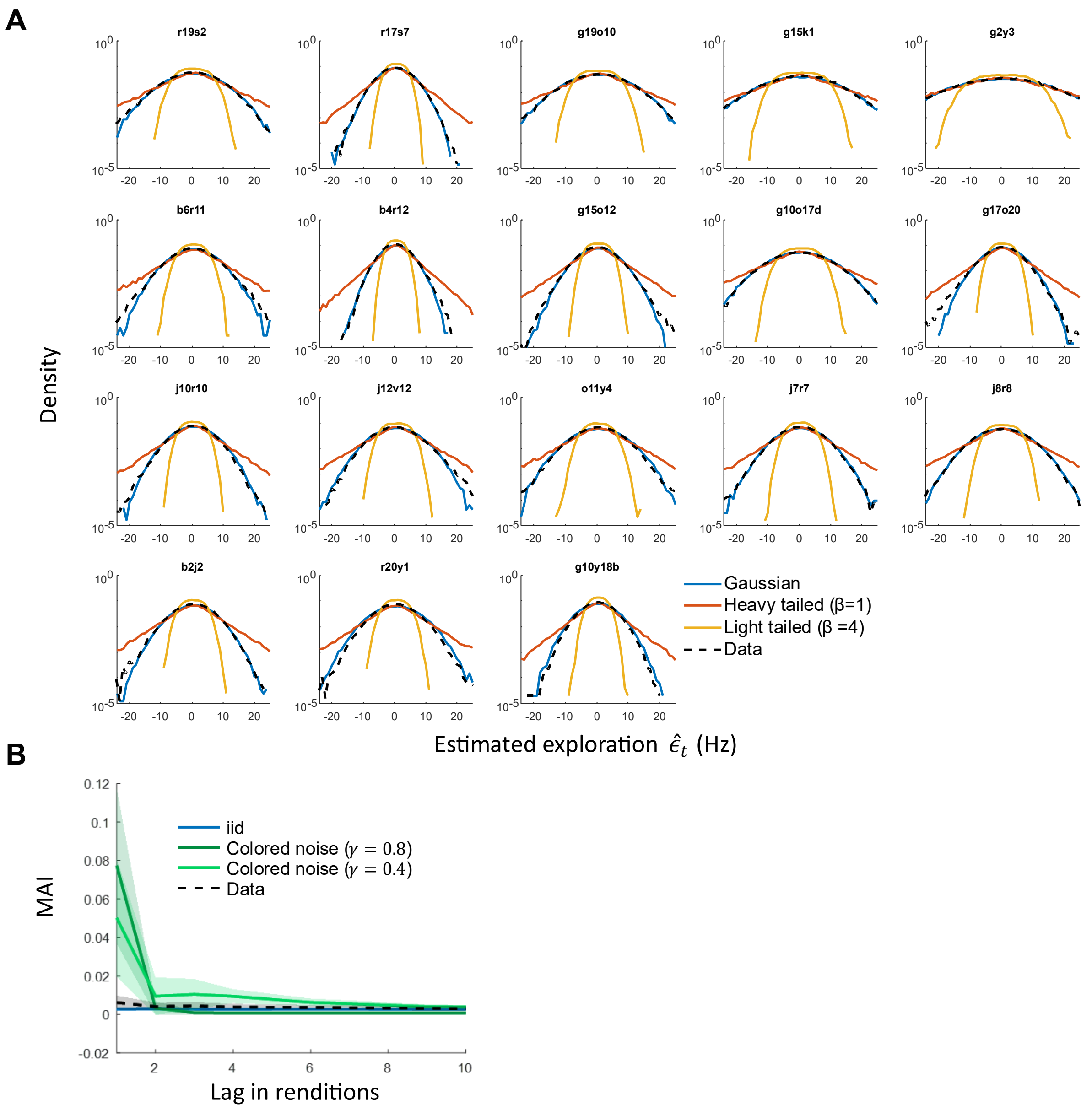

Figure S2: Estimating exploration from simulated data confirms that explorations are Gaussian and iid, related to Figure 2. (A) Pitch trajectories were generated with exploration drawn from a generalized normal distribution that was Gaussian (β = 2), heavy-tailed (β = 1), or light-tailed (β = 4). Although the Kalman filter assumes Gaussian variability in all cases, it does not impose this statistic and the estimation procedure accurately recovered the underlying exploration statistics of $\hat{\epsilon}_t$ (Gaussian in blue, heavy-tailed in red, and light tailed in yellow). The density distribution of explorations estimated from birds' data (black dashed line) best matches the Gaussian case (shown are all birds separately), showing that we would have recovered non-Gaussian statistics if birds displayed them, supporting our assumption of Gaussian exploration. (B) Same as (A) but pitch synthetic explorations were drawn from a distribution $\epsilon_t = \gamma\epsilon_{t-1} + n_t$ with n_t iid Gaussian noise. Explorations ϵ_t were either iid ($\gamma = 0$) or temporally correlated (with $\gamma = 0.4$ or $\gamma = 0.8$; colored noise explorations were normalized so their variance matched the iid case). Again, although the Kalman filter assumes iid variability, it does not impose it and the estimation procedure accurately recovered

the underlying temporal structure of the colored noise at lag 1 ($\gamma = 0.4$ light green, $\gamma = 0.8$ dark green). The mutual average information (MAI) of explorations estimated from birds' data (black dashed line) most closely matches the iid case (blue), confirming Gaussian iid exploration and that we would recover temporal correlations if birds displayed them. The lines and shaded area correspond to the means and standard deviations across N=18 birds.

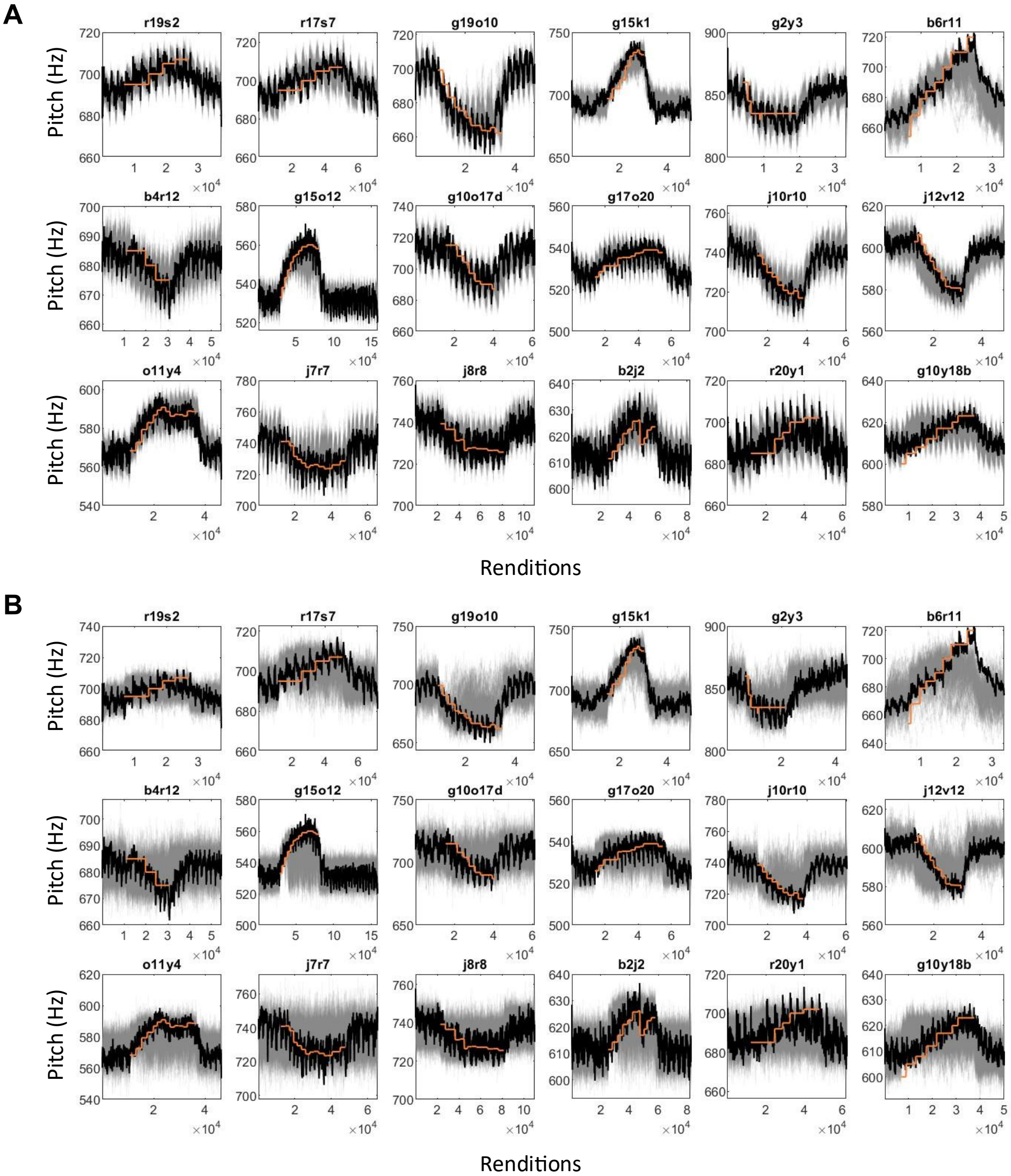

Figure S3: Model simulations of the latent and classical RL model, related to Figure 3. (A) Latent RL: The observed smoothed pitch trajectory (black line) lies mostly within 100 simulated smoothed trajectories using the basic latent RL model (gray) and the best fit parameters for each bird, N=18 birds. (B) Classcial RL: The observed smoothed pitch trajectory (black line) rises slowly over the course of several days whereas the smoothed simulated trajectories (gray) suggest quick learning in the beginning. Simulations are done by running the basic RL model equations, randomly sampling the noise components and using the best fit parameters for each bird, N=18 birds.

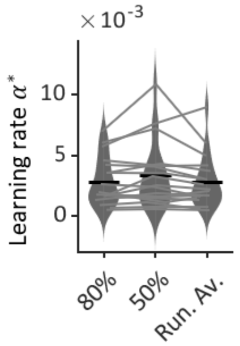

Figure S4: **The best-ft learning rate α^* does not depend on the threshold update rules, related to Figure 4.** The best-fit learning rate does not depend on the update rule of the threshold. We simulated 19 learning trajectories using the best fit parameters of each bird and three different update rules: the threshold is set to the 80th percentile of the previous day (80%), to the median of the previous day (50%), or to the running average over the past 20 renditions.

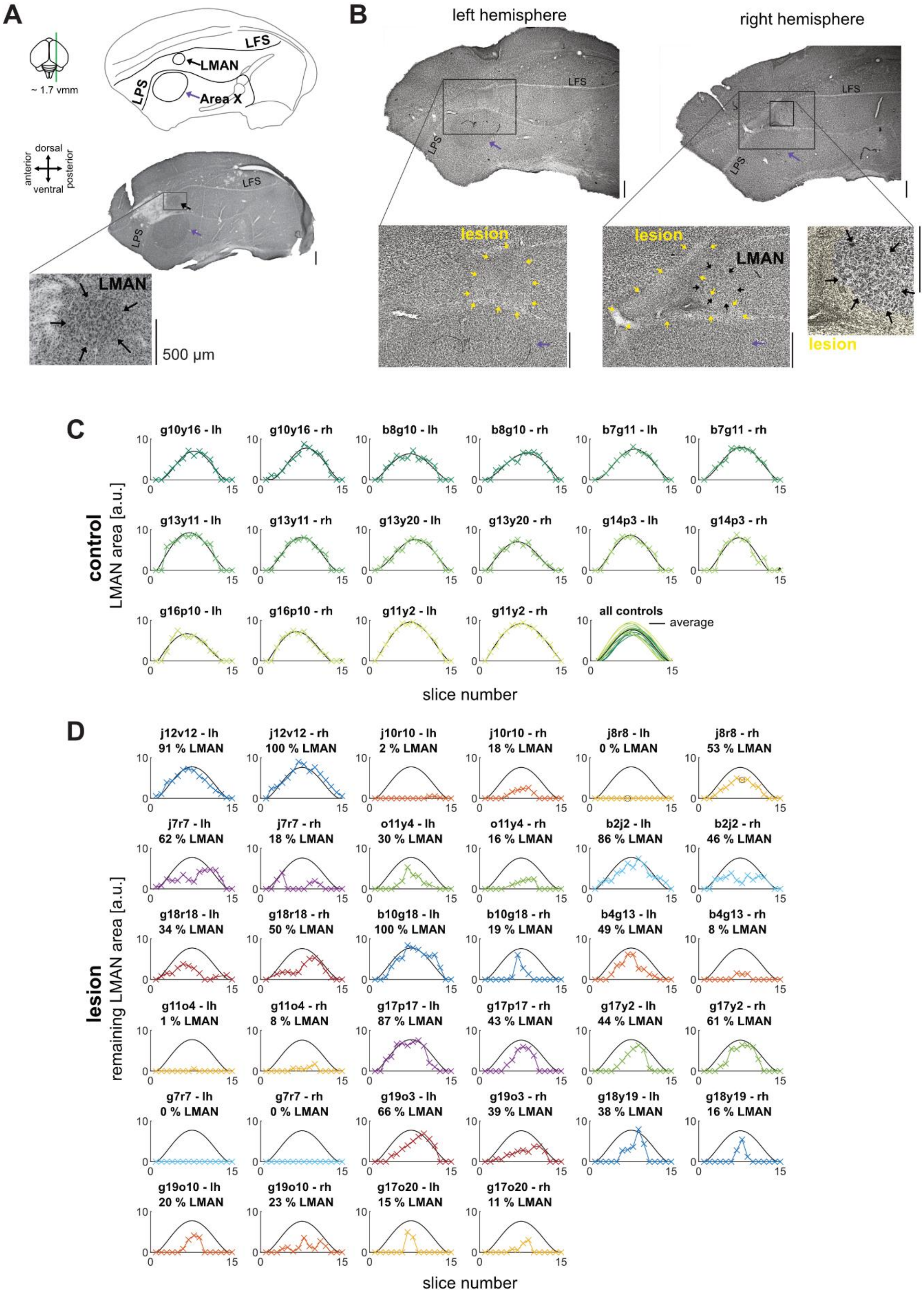

Figure S5: Quantification of lesion extent, related to Figure 5. (A) Schematic of sagittal brain section approximately 1.7 mm lateral to the midline (top), illustrating LMAN's location dorsal to Area X and between the lamina frontalis superior (LFS) and the lamina pallio-subpallialis (LPS). Relevant structures are delimited or indicated by thick lines. In Nissl-stained sagittal brain sections (bottom),

Area X (purple arrow) and LMAN (black arrow) stand out due to their high neural density and large cell nuclei (zoomed-in area delimited by grey rectangle). (B) Sagittal brain slices of lesioned brains (top). Remaining unlesioned parts of LMAN (black arrows) were quantified in magnified pictures (bottom, see Methods). In this example section in the left hemisphere (left), LMAN was absent, indicative of a complete lesion (yellow arrows indicate the lesion boundary). In the right-hemisphere example section (right), the injection was too anterior, missing 59% of LMAN. (C and D) *Shown is the (remaining) LMAN extent in each hemisphere of the 8 controls (C) and 17 lesioned birds (D). The LMAN extent is plotted as the area in consecutive sagittal brain slices. The LMAN extent in each control bird was fitted with a polynomial function (black lines). The average fit (black line) is shown on the bottom right. LMAN extent in lesioned brains (colored crosses and lines). For comparison, in each hemisphere, we also plotted the control-averaged LMAN extent (aligned to best fit the remainder of LMAN). The sections shown in Supplementary Figure S5B are indicated by black circles. In three lesioned birds (b8g10, b7g11, g11y2, included in Figure 5), LMAN was missed completely, and they were used here as control birds to quantify LMAN volume. We did not take g7r7 as a control bird because the lesion touched LMAN but we excluded it from Figure 5G because the estimated lesion extent is 0% in both hemispheres.*

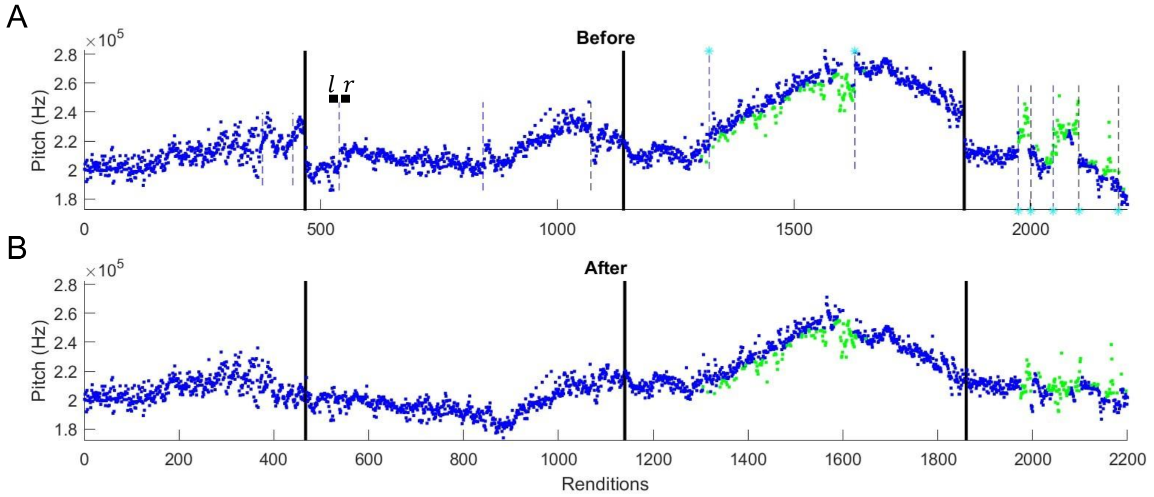

Figure S6: **Human pitch steps, related to Figure 6**. (A) Steps are detected at utterances where the left pitch and the right pitch differ by more than 5% (the left and right windows of a step are indicated as horizontal thick bars and labelled l and r). Upward steps are indicated by blue dashed lines and downward steps by black dashed lines. Pitch steps preceded by WN are indicated by a cyan asterisk, shown at the top for low-pitch WN contingency and at the bottom for high-pitch contingency. Utterances followed by WN are indicated in green. Session boundaries are indicated by thick vertical lines. In the shown time period, the subject exhibits 5 pitch steps without WN and 7 pitch steps preceded by WN. (B) Due to the iterative method of detecting pitch steps, the pitch trajectory looks smoother after step removal.

Supplementary Methods

Parameter and state estimation

We compute the best fit parameters $\theta_i = \{(1-\tau), D_1, \ldots, D_{N_d+1}, O_1, \ldots, O_{N_h}\}$ and variances $(\sigma_\varepsilon^2, \sigma_\epsilon^2)$ governing the baseline period and the parameters $\Theta_i = \{(1-\delta), \alpha\}$ and $(\sigma_\eta^2, \sigma_\epsilon^2)$ of the latent learner to behavioral data using the iterative expectation maximization (EM) algorithm together with the Kalman filter for state estimation [1,2]. The EM objective is to fit the optimal parameters by maximizing the log-likelihood of the data, given the model parameters.

To fit the parameters of the linear dynamical system, we use a modification of the method introduced by Ghahramani and Hinton.[1,2] They estimate the parameters of the following linear dynamical system:

$$z_t = Hx_t + g_t$$

$$x_t = Ax_{t-1} + q_t,$$

where $t = 1,2,\ldots,T$ denotes a discrete time index. Only the output z_t is observable and is assumed to be a linear function of the hidden state x_t. The state x_t depends linearly on the previous state x_{t-1}. Both the output noise g_t and the state noise q_t are zero-mean Gaussian random variables with covariance matrices G and Q, respectively[1]. The hidden state x_t is estimated using a Kalman filter and the model variables (state transition matrix A, output matrix H, and the covariance matrices G and Q) are estimated iteratively using the expectation maximization (EM) algorithm.

We make two modifications of compared to Ghahramani and Hinton.[1] First, our system contains a time dependent input u_t that depends both on the time of the day of rendition t, the position in the song bout, and possibly the pitch/syllable duration of the last rendition z_{t-1}, which renders our model compatible with.[3] Second, instead of a fixed transition matrix A, our transition matrix C_t is time dependent and has several entries that are either linked to other entries or are forced to be zero. The following sections describe the parameter estimation, differences from Ghahramani & Hinton are mentioned where applicable.

Data preparation

The standard EM algorithm works well when the time-dependent input u_t depends on explicit variables[3] such as the time of renditions t and $t-1$. However, in our case there is also a time-dependent transition matrix C_t with components that are either zero (e.g. $(C^1)_{kl} = 0$ except if $k = l = 1$) or are shared among model components (e.g. $(C_t^2)_{13} = -(C_t^2)_{15}$ during the feedback period). To deal with this situation, we used the method described in [1–3] that applies when the following holds:

1. Q is diagonal
2. C_t can be written as $C_t = C_t^0 + \sum_i \Theta_i C_t^i$ with $\left(C_t^i\right)_{m,n} = 0$ for $m \neq m_i$, where m_i is the row of C_t in which parameter Θ_i is located

Before estimating the parameters, we perform mean subtraction (we subtract $\bar{p} = \frac{1}{T_b}\sum_{t=1}^{T_b} p_t$ from the measured pitch values, where T_b marks the end of baseline. This is not crucial but helps to avoid numerical instabilities arising from the large ratio of 10^6 between the smallest parameter (e.g. $\delta \approx$

[1] Note that in the literature the term g_t is often referred to as r_t.

0.001) and the largest parameter ($p^* \approx 1000$ Hz). Thus, in practice, the estimated target pitch is $\hat{p}^* = \bar{p} + \frac{1}{T_b}\sum_{t=1}^{T_b} \hat{d}_t$, which we keep constant during the feedback period.

We further add the constraint that the history dependence averages to zero and does not add an overall pitch bias; we enforce this constraint after every EM iteration by transferring the non-zero history average to the circadian pattern: $D_i \to D_i + \frac{1}{T_b}\sum_{t=1}^{T_b} o_t$ and $O_i \to O_i - \frac{1}{T_b}\sum_{t=1}^{T} o_t$.

Detailed Equations

We estimate parameters φ iteratively using the expectation maximization (EM) algorithm that maximizes the log-likelihood $\mathrm{L}(\varphi) = \ln p(Z_T|\varphi)$ of observing the (pitch) sequence $Z_T = \{z_1, \dots, z_T\}$. The EM algorithm consists of two iterative steps. Briefly, in the expectation step (E-step), the expected hidden state sequence is computed given all the observations up to the given time step and the set of current model parameters, this computation is based on the forward algorithm. The forward algorithm is followed by the backward algorithm that yields the expected hidden state sequence based on all observations. Then, in the maximization step of the EM algorithm (M-step), the expected hidden state sequence is used to find model parameters that maximize the current log-likelihood. By iteratively estimating the best hidden state sequence and the best model parameters via the E and M steps, the log-likelihood of observing the data given the model parameters monotonically increases.

More specifically, we split the parameter estimation into three parts:

1. Log-likelihood: Estimating the log-likelihood $\mathrm{L}(\varphi) = \ln p(Z_T|\varphi)$ of observing the output sequence Z_T given the model parameters φ
2. E-Step:
   a. Forward algorithm (Kalman filter): Estimating the hidden state sequence $\{x_t^t\}$ given the model parameters φ and all observations up to time t, where $x_t^\tau \equiv E[x_t|Z_\tau, \varphi]$ with $x_1^0 = v$.
   b. Backward algorithm: Estimating the hidden state sequence $\{x_t^T\}$ given the model parameters and all observations up to final time T.
3. M-step: find the model parameters φ that maximize the log likelihood $\mathrm{L}(\varphi) = \ln p(Z_T|\varphi)$.

To simplify the notation, let us introduce the variable $y_{t-1} = \begin{pmatrix} u_t \\ x_{t-1} \end{pmatrix}$ that is a combination of both the state x_{t-1} and the input u_t. We further introduce the transition matrix $C_t = [B_t \quad A_t]$. Using this notation, we can write:

$$z_t = Hx_t + g_t$$

$$x_t = A_t x_{t-1} + B_t u_t + q_t = C_t y_t + q_t.$$

We further define $X_t = (x_1, \dots, x_t)$ and $Y_t = (y_1, \dots, y_t) = (x_1, u_1, \dots, y_t, u_t)$ analogous to Z_t.

1 Log-likelihood estimation

For the M step we need to know the log-likelihood $\mathrm{L}(\varphi) = \ln p(Z_T|\varphi)$. If $\hat{y}_{t-1}$ is the expected state at time $t-1$, then at time t, the expected state is $\hat{x}_t = C_t\hat{y}_{t-1} = A_t\hat{x}_{t-1} + B_t u_t$. The initial state distribution $p(x_1)$ satisfies $p(x_1|v, V) = N(v, V)$, where $v = \hat{x}_1$ is the expected initial state and V the initial covariance matrix.

We compute the likelihood of the observation sequence by recursion over t. The likelihood of observing z_1 given the model parameters satisfies:

$$p(z_1|\varphi) = \int p(z_1|x_1, \varphi)p(x_1|v, V, u_1)dx_1,$$

where $p(z_1|x_1, \varphi) = N(Hx_1, G)$. Then, the probability at time t can be derived recursively using Bayes' rule:

$$p(Z_t|\varphi) = p(Z_{t-1}|\varphi)p(z_t|Z_{t-1}, \varphi).$$

The likelihood $p(z_t|Z_{t-1}, \varphi) = \int p(z_t|x_t, \varphi)p(x_t|Z_{t-1}, u_t, \varphi)\, dx_t$ with

$$p(z_t|x_t, \varphi) = N(Hx_t, G)$$

$$p(x_t|Z_{t-1}, u_t, \varphi) = N(\hat{x}_t, \bar{P}_t)$$

where $\bar{P}_t = A_t\bar{P}_{t-1}{A_t}^T + Q$ is the covariance matrix of the state estimate, is given by:

$$p(z_t|Z_{t-1}, \varphi) = \; N(H\hat{x}_t, G + \bar{P}_t H^2).$$

Finally, the likelihood of the entire output sequence given model parameters satisfies:

$$p(Z_T|\varphi) = p(z_1|\varphi)\prod_{t=2}^{T} p(z_t|Z_{t-1}, \varphi),$$

and so we find for the log-likelihood:

$$L(\varphi) = -\sum_{t=1}^{T}\left(\frac{1}{2}[z_t - H\hat{x}_t]^T[G + H\bar{P}_t H^T]^{-1}[z_t - H\hat{x}_t]\right) + \frac{T}{2}\ln|G + H\bar{P}_t H^T| + \frac{Tp}{2}\log 2\pi$$

with p being the dimension of z_t.

2 Forward and backward algorithms

Given the model parameters φ and the sequence of observations Z_T, we are looking for the most likely hidden state sequence $\hat{X}_T$ that gave rise to the observations. In other words, we are looking for the hidden state sequence X_T that maximizes the probability $p(X_T|Z_T, \varphi)$ given the observation sequence:

$$\hat{X}_T = \underset{X_T}{\operatorname{argmax}}\, p(X_T|Z_T, \varphi).$$

The forward algorithm provides part of the solution, namely the distribution $p(x_t|Z_t, \varphi)$ of the state x_t given all observations Z_t up to time t. By defining $y_t^\tau \equiv \binom{u_t}{x_t^\tau} = E[y_t|Z_\tau, \varphi]$ and $V_t^\tau \equiv \mathrm{Var}[x_t|Z_\tau, \varphi]$ with $V_1^0 = V$ (the initial covariance matrix), the forward recursion can be written as:

$$x_t^{t-1} = C_t y_{t-1}^{t-1}$$

$$V_t^{t-1} = A_t V_{t-1}^{t-1} A_t' + Q$$

$$K_t = V_t^{t-1} H'(HV_t^{t-1}H' + G)^{-1}$$

$$x_t^t = x_t^{t-1} + K_t(z_t - Hx_t^{t-1})$$

$$V_t^t = (1 - K_t H)V_t^{t-1}.$$

We further introduce the following simplifying notation:

$$\hat{x}_t \equiv x_t^T = E[x_t|Z_T]$$

$$P_t \equiv E[x_t x_t'|Z_T] = V_t^T + x_t^T x_t^{T\prime}.$$

Note that in contrast to the classical Kalman filter, $\hat{x}_t$ does not only depend on the observations up to time t but depends on all past and future observations Z_T. For the maximization, we need the expectation values ($\hat{x}_t$ and P_t) given all the past and future observations Z_T. For $t = T$, this corresponds trivially to the expectation values from the forward algorithm, for example:

$$\hat{x}_T \equiv E[x_T|Z_T] = \hat{x}_T^T.$$

According to Ghahramani and Hinton and following [2], the backward recursion is given by:

$$J_{t-1} = V_{t-1}^{t-1} A_t'(V_t^{t-1})^{-1}$$

$$x_{t-1}^T = x_{t-1}^{t-1} + J_{t-1}(x_t^T - C_t x_{t-1}^{t-1})$$

$$V_{t-1}^T = V_{t-1}^{t-1} + J_{t-1}(V_t^T - V_t^{t-1})J'_{t-1}.$$

Furthermore, we also need the following recursive quantity

$$P_{t,t-1} \equiv V_{t,t-1}^T + x_t^T x_{t-1}^T{}'$$

with

$$V_{t-1,t-2}^T = V_{t-1}^{t-1} J'_{t-2} + J_{t-1}\big(V_{t,t-1}^T - A_t V_{t-1}^{t-1}\big)J'_{t-2},$$

which can be initiated with $V_{T,T-1}^T = (1 - K_T H)A_T V_{T-1}^{T-1}$.

Thus, we now have the most likely hidden state sequence X_T^T given the model parameters φ and the sequence of observations Z_T. In other words, X_T^T maximizes the likelihood $p(X_T|Z_T, \varphi)$.

3. The learning problem: M-step

Given the sequence of observations Z_T, the aim is to find the model parameters φ that maximize the log likelihood $\ln p(Z_t|\varphi)$. According to the EM algorithm, we can achieve this by iterating over models $\varphi^{(1)}, \ldots, \varphi^{(k)}, \varphi^{(k+1)}$ where $\varphi^{(k+1)}$ maximizes an auxiliary function that serves as lower bound of the log likelihood:

$$\varphi^{(k+1)} = \underset{\psi}{\operatorname{argmax}} \int p\big(X_T|Z_T, \varphi^{(k)}\big) \ln p(X_T, Z_T|\psi)\, dx_1 \ldots dx_T$$

Due to the Markov property implicit in the model, we can write the joint probability of observations Z_T and states X_T as

$$p(X_T, Z_T|\psi) = p(x_1) \prod_{t=2}^{T} p(x_t|x_{t-1}) \prod_{t=1}^{T} p(z_t|x_t)$$

with

$$p(z_t|x_t) = \exp\left\{-\frac{1}{2}[z_t - Hx_t]'G^{-1}[z_t - Hx_t]\right\}(2\pi)^{-p/2}|G|^{-1/2}$$

$$p(x_t|x_{t-1}) = \exp\left\{-\frac{1}{2}\left[x_t - C_t\begin{pmatrix}u_t\\x_{t-1}\end{pmatrix}\right]'Q^{-1}\left[x_t - C_t\begin{pmatrix}u_t\\x_{t-1}\end{pmatrix}\right]\right\}(2\pi)^{-k/2}|Q|^{-1/2}$$

$$p(x_1) = \exp\left\{-\frac{1}{2}[x_t - v]'{V_1}^{-1}[x_1 - v]\right\}(2\pi)^{-k/2}|V_1|^{-1/2}.$$

Therefore, the joint log probability is a sum of quadratic terms,

$$\begin{aligned}\ln p(X_T, Z_T|\psi) = &-\sum_{t=1}^{T}\frac{1}{2}[z_t - Hx_t]'G^{-1}[z_t - Hx_t] - \frac{T}{2}\ln|G| \\ &-\sum_{t=1}^{T}\frac{1}{2}[x_t - C_t y_{t-1}]'Q^{-1}[x_t - C_t y_{t-1}] - \frac{T-1}{2}\ln|Q| \\ &-\frac{1}{2}[x_1 - v]'{V_1}^{-1}[x_1 - v] - \frac{1}{2}\ln|V_1| - \frac{T(p+k)}{2}\ln 2\pi.\end{aligned}$$

For simplicity, we introduce the abbreviated notation $\int p\left(X_T|Z_T, \varphi^{(k)}\right) f(X_T) dx_1 \dots dx_T \equiv \mathrm{E}[f(X_T)|Z_T]$. The parameters of this system are $\vartheta_j = \left\{\theta_{i=1,\dots,c}, G, Q, v, V_1\right\}$ where θ_i are the parameters describing the transition matrix C_t. Each parameter must be re-estimated by taking the corresponding partial derivative, setting it to zero, and solving for the parameters:

$$\frac{\partial}{\partial\vartheta_j}\mathrm{E}[\ln p(X_T, Z_T|\vartheta)|Z_T] = 0$$

For $|G| > 0$ and $|Q| > 0$, the log likelihood is both continuous and differentiable. Thus, we can first take the derivative and then compute the expectation:

$$\leftrightarrow \mathrm{E}\left[\left(\frac{\partial}{\partial\vartheta_j}\ln p(X_T, Z_T|\vartheta)\right)|Z_T\right] = 0.$$

In the following, we show how these expectations can be computed iteratively.

Initial state mean and covariance:

The initial state is described by its mean v and covariance V_1 and can be estimated according to:

$$\frac{\partial}{\partial v}\ln p(X_T, Z_T|\vartheta) = [x_1 - v]{V_1}^{-1},$$

which can be solved for the mean initial state:

$$v^{(k+1)} = \hat{x}_1.$$

Analogously we can solve

$$\frac{\partial}{\partial {V_1}^{-1}}\ln p(X_T, Z_T|\vartheta) = \frac{1}{2}V_1 - \frac{1}{2}[x_1 - v][x_1 - v]'$$

to find the initial covariance

$${V_1}^{(k+1)} = P_1 - \hat{x}_1\hat{x}_1'.$$

State noise covariance:

In contrast to the EM algorithm for linear dynamical models introduced by Shumway and Stoffer, the state noise covariance matrix in our model is only non-zero on the diagonal. In this case, maximizing the log likelihood with respect to the individual matrix entries $q_{i,j}$ is equivalent to maximizing with respect to the whole matrix Q:

$$\mathrm{E}\left[\left(\frac{\partial}{\partial q_{i,j}} \ln p(X_T, Z_T|\vartheta)\right)|Z_T\right] = 0 \quad \forall i,j \;\Leftrightarrow\; \mathrm{E}\left[\left(\frac{\partial}{\partial Q} \ln p(X_T, Z_T|\vartheta)\right)|Z_T\right] = 0$$

Thus, we can first derive the M step for a general state covariance matrix $\tilde{Q}$ and then set the off-diagonal matrix elements to zero to get the updated covariance matrix Q for our specific model:

$\frac{\partial}{\partial Q^{-1}} \ln p(X_T, Z_T|\vartheta) = 0$ yields the optimal covariance matrix

$$\tilde{Q}^{(k+1)} = \frac{1}{T-1}\sum_{t=2}^{T}[x_t - C_t y_t][x_t - C_t y_t]' =$$

$$= \frac{1}{T-1}\sum_{t=2}^{T}\left(P_t - C_t^{(k+1)}P_{t-1,t} - P_{t,t-1}C_t^{(k+1)'} + C_t^{(k+1)}P_{u,t-1}C_t^{(k+1)'}\right)$$

The updated model state noise covariance matrix $Q^{(k+1)}$ is therefore a diagonal matrix with diagonal components:

$$q_{i,i}^{(k+1)} = \tilde{q}_{i,i}^{(k+1)},$$

since constraining the off-diagonal components to zero has no effect on the optimal diagonal components.

Note that $Q^{(k+1)}$ depends on the new state transition matrix $C_t^{(k+1)}$ and thus $Q^{(k+1)}$ can be computed only after re-estimating the model parameters of the state transition matrix which luckily can be achieved without knowing the new state noise covariance.

Output noise covariance:

In our model, the output is one dimensional and thus the covariance matrix is trivially diagonal. In case of a higher dimensional output, the optimal noise covariance $G^{(k+1)}$ has the same diagonal as the matrix $\tilde{G}$ that satisfies

$$\frac{\partial}{\partial G^{-1}} \ln p(X_T, Z_T|\vartheta) = 0$$

and so

$$\tilde{G} = \frac{1}{T}\sum_{t=1}^{T}(z_t z_t' - 2H\hat{x}_t z_t + HP_t H').$$

State transition matrix:

The general form of the state transition matrix can be written as $C_t = C_t^0 + \sum_i \theta_i C_t^i$, where θ_i are the model parameters we are looking for and C_t^i is a rigid matrix defining the model structure.

Taking the derivative of the log-likelihood yields

$$\frac{\partial}{\partial\theta_i}\ln p(X_T, Z_T|\vartheta) = \sum_{t=2}^{T}\mathrm{tr}\left[Q^{-1}(x_t - C_t y_{t-1})y_{t-1}{}' C_t^{i}{}'\right]$$
$$= \sum_{t=2}^{T}\mathrm{tr}\left[Q^{-1}\left(x_t - \left(C_t^0 + \sum_j \theta_j C_t^j\right)y_{t-1}\right)y_{t-1}{}' C_t^{i}{}'\right].$$

In general, this last expression depends on Q in a non-trivial way and therefore the equations for Q and θ_i are hard to separate and solve analytically. However, in the special case where 1: Q is diagonal and 2: C_t^i has non-zero entries only in one single row m_i, we can get rid of Q when setting the above equation to zero.

The first requirement is satisfied because we defined our models that way. The second requirement is satisfied only in models as ours in which parameters apply only to single model components (are not expressed in more than one equation).

Assumptions (constraints): 1) $q_{m,n} = 0$ for $m \neq n$ (Q is diagonal)

2) $\left(C_t^i\right)_{m,n} = 0$ for $m \neq m_i$

If the conditions 1 and 2 are satisfied, we can simplify the derivative (for a proof, see[4]).

$$0 = \sum_{t=2}^{T}\mathrm{tr}\left[\left(x_t - \left(C_t^0 + \sum_j \theta_j C_t^j\right)y_{t-1}\right)y_{t-1}{}' C_t^{i}{}'\right]$$

and thus,

$$\sum_{t=2}^{T}\mathrm{tr}\left[(x_t - C_t^0 y_{t-1})y_{t-1}{}' C_t^{i}{}'\right] = \sum_j \theta_j \sum_{t=2}^{T}\mathrm{tr}\left[C_t^j y_{t-1} y_{t-1}{}' C_t^{i}{}'\right].$$

The left side of the equation only depends on i whereas the right side depends on i and j. The θ_j maximizing the log likelihood has to satisfy the above equation for every i. We can rewrite this in matrix form:

$$F = E * \boldsymbol{\theta}^{(k+1)},$$

Where '*' denotes matrix multiplication, $\boldsymbol{\theta}^{(\boldsymbol{k}+\mathbf{1})}$ is a vector containing all the parameter $\theta_j^{(k=1)}$, and F and E are defined as:

$$f_i = \mathrm{tr}\left[\sum_{t=2}^{T}\left(P_{t,t-1} - C_t^0 P_{u,t-1}\right)C_t^{i}{}'\right]$$

$$e_{i,j} = \mathrm{tr}\left[\sum_{t=2}^{T} C_t^j P_{u,t-1} C_t^{i}{}'\right]$$

with

$$P_{u,t-1} \equiv E[y_{t-1}y_{t-1}{}'|Z_T].$$

This is a simple system of linear equations that can be solved for the model parameters $\boldsymbol{\theta}^{(k+1)}$.

Theoretical learning speeds and limits

Maximum learning could be achieved in the absence of non-ideal behavioral variability and by adapting the threshold to the current mean pitch $\theta = p^* + b_t$. In this case, the expected correlation between reward and exploration is

$$\langle R_t \cdot \epsilon_t \rangle = \int_{-\infty}^{0} \frac{-\epsilon}{\sigma_\epsilon \sqrt{2\pi}} e^{-\frac{\epsilon^2}{2\sigma_\epsilon^2}} d\epsilon = \frac{\sigma_\epsilon}{\sqrt{2\pi}},$$

Assuming the bird receives white noise for low pitched renditions. Thus, in this ideal scenario without non-ideal inaccessible variability, the bias would increase until it reaches $b^{\max} = \frac{\alpha\sigma_\epsilon}{\delta\sqrt{2\pi}}$ and thus the maximum shift a bird could achieve scales with both the learning rate α and the exploration standard deviation σ_ϵ.

In the presence of inaccessible variability V_t and arbitrary threshold θ, the expected correlation between reward and exploration becomes:

$$\langle R_t \cdot \epsilon_t \rangle = \int_{-\infty}^{\theta-(b_t+V_t)} \frac{-\epsilon}{\sigma_\epsilon \sqrt{2\pi}} e^{-\frac{\epsilon^2}{2\sigma_\epsilon^2}} d\epsilon = \frac{\sigma_\epsilon}{\sqrt{2\pi}} e^{-\frac{(b_t+V_t-\theta)^2}{2\sigma_\epsilon^2}}.$$

Thus, learning is expected to decrease with increasing non-ideal variability V_t.

Varia

Latent RL can be seen as a prerequisite of more advanced motor control theories where the optimal strategy in the face of uncertainty is to allow variability in redundant (task-irrelevant) dimensions[5] – latent RL can help to identify these dimensions, since latent RL implements a local search for more reward using small perturbations. The use of perturbations as a means to probe a system is known to estimations of Lyapunov exponents.[6]

In previous models it was not possible to estimate the exploration variance because the latter (i.e., the variability of motor output exploitable by the learning rule) was assumed to be proportional to learning speed[7,8] and therefore could be estimated when no learning happens (e.g. during free behavior). Thus, our model breaks the symmetry between rapid learning from a weak source of variability and slow learning from a strong source.

It will be important to gain further insights into the apparent randomness of animal behavior[9,10].

Latent RL can be seen as a rigorous recipe for detrending, often seen as a questionable procedure. Among all preprints containing the word stems 'detrend' or 'trend removal' published on Biorxiv in the years 2022 and 2023, 87% also used one of the following words: 'animal', 'ethology', or 'behavior'. Thus, detrending is mainly practiced in neuroethology where latent RL can be impactful.

Latent RL is not to be confused with using a latent model to perform reinforcement learning. Latent models have been used in reinforcement learning to describe causes in the environment capable of predicting the presence or absence of a reinforcer.[11] These models put the emphasis on world models, which are absent in latent RL.

Bibliography

1. Ghahramani, Z., and Hinton, G.E. (1996). Parameter Estimation for Linear Dynamical Systems. University of Toronto technical report CRGTR962 *6*, 1–6. https://doi.org/10.1080/00207177208932224.

2. Shumway, R.H., and Stoffer, D.S. (1982). An approach to time series smoothing and forecasting using the EM algorithm. Journal of Time Series Analysis *3*, 253–264. https://doi.org/10.1111/j.1467-9892.1982.tb00349.x.

3. Cheng, S., and Sabes, P.N. (2006). Modeling sensorimotor learning with linear dynamical systems. Neural computation *18*, 760–793. https://doi.org/10.1162/neco.2006.18.4.760.

4. Zai, A.T. (2019). Inferring the trial-by-trial structure of pitch reinforcement learning in songbirds. ETH Zurich. https://doi.org/10.3929/ethz-b-000382074.

5. Todorov, E., and Jordan, M.I. (2002). Optimal feedback control as a theory of motor coordination. Nature Neuroscience *5*, 1226–1235. https://doi.org/10.1038/nn963.

6. Balcerzak, M., Sagan, T., Dabrowski, A., and Stefanski, A. (2020). Fast and simple Lyapunov Exponents estimation in discontinuous systems. Eur. Phys. J. Spec. Top. *229*, 2167–2181. https://doi.org/10.1140/epjst/e2020-900275-x.

7. Therrien, A.S., Wolpert, D.M., and Bastian, A.J. (2016). Effective reinforcement learning following cerebellar damage requires a balance between exploration and motor noise. Brain *139*, 101–114. https://doi.org/10.1093/brain/awv329.

8. Therrien, A.S., Wolpert, D.M., and Bastian, A.J. (2018). Increasing motor noise impairs reinforcement learning in healthy individuals. eNeuro *5*. https://doi.org/10.1523/ENEURO.0050-18.2018.

9. Ferdous, M.J., Reynolds, A.M., and Cheng, K. (2018). Distinguishing between apparent and actual randomness: a preliminary examination with Australian ants. Behav Ecol Sociobiol *72*, 113. https://doi.org/10.1007/s00265-018-2527-1.

10. Page, Suzanne, and Neuringer, Allen (1985). Variability is an operant. Journal of Experimental Psychology: Animal Behavior Processes, *11*.

11. Gershman, S.J., Norman, K.A., and Niv, Y. (2015). Discovering latent causes in reinforcement learning. Current Opinion in Behavioral Sciences *5*, 43–50. https://doi.org/10.1016/j.cobeha.2015.07.007.
